# Comparative transcriptomic analysis provides insights into the genetic networks regulating oil differential production in oil crops

**DOI:** 10.1186/s12915-024-01909-x

**Published:** 2024-05-13

**Authors:** Jinwen Chen, Yan Hu, Ting Zhao, Chujun Huang, Jiani Chen, Lu He, Fan Dai, Shuqi Chen, Luyao Wang, Shangkun Jin, Tianzhen Zhang

**Affiliations:** 1 Department of Agronomy, College of Agriculture and Biotechnology, Zhejiang University, Hangzhou, 310029 Zhejiang China; 2 Hainan Institute of Zhejiang University, Sanya, 572025 Hainan China; 3 State Key Laboratory of Crop Genetics and Germplasm Enhancement, College of Agriculture, Nanjing Agricultural University, Nanjing, 210095 Jiangsu China

**Keywords:** Network alignment, Seed oil synthesis, Coexpression networks, Comparative transcriptomic, Differential expression analysis, Seed storage, Acyl-lipid metabolism, Different species

## Abstract

**Background:**

Plants differ more than threefold in seed oil contents (SOCs). Soybean (*Glycine max*), cotton (*Gossypium hirsutum*), rapeseed (*Brassica napus*), and sesame (*Sesamum indicum*) are four important oil crops with markedly different SOCs and fatty acid compositions.

**Results:**

Compared to grain crops like maize and rice, expanded acyl-lipid metabolism genes and relatively higher expression levels of genes involved in seed oil synthesis (SOS) in the oil crops contributed to the oil accumulation in seeds. Here, we conducted comparative transcriptomics on oil crops with two different SOC materials. In common, *DIHYDROLIPOAMIDE DEHYDROGENASE*, *STEAROYL-ACYL CARRIER PROTEIN DESATURASE*, *PHOSPHOLIPID:DIACYLGLYCEROL ACYLTRANSFERASE*, and oil-body protein genes were both differentially expressed between the high- and low-oil materials of each crop. By comparing functional components of SOS networks, we found that the strong correlations between genes in “glycolysis/gluconeogenesis” and “fatty acid synthesis” were conserved in both grain and oil crops, with *PYRUVATE KINASE* being the common factor affecting starch and lipid accumulation. Network alignment also found a conserved clique among oil crops affecting seed oil accumulation, which has been validated in *Arabidopsis*. Differently, secondary and protein metabolism affected oil synthesis to different degrees in different crops, and high SOC was due to less competition of the same precursors. The comparison of *Arabidopsis* mutants and wild type showed that *CINNAMYL ALCOHOL DEHYDROGENASE 9*, the conserved regulator we identified, was a factor resulting in different relative contents of lignins to oil in seeds. The interconnection of lipids and proteins was common but in different ways among crops, which partly led to differential oil production.

**Conclusions:**

This study goes beyond the observations made in studies of individual species to provide new insights into which genes and networks may be fundamental to seed oil accumulation from a multispecies perspective.

**Supplementary Information:**

The online version contains supplementary material available at 10.1186/s12915-024-01909-x.

## Background

Oil crops provide an abundant and renewable source of vegetable oil and protein for the human diet, animal feeds, and industrial production. According to the oilseed production, supply, and distribution report from the US Department of Agriculture (data available in April 2022), over the past 5 years, worldwide vegetable oil consumption has been up to 209 million metric tons, growing by 9.1%, with palm (35.3%), soybean (28.5%), rapeseed (13.9%), and sunflower seed (9.5%) taking a larger share. Therefore, to meet the rapid growth of food demand and nonfood utilization, enhancing oil productivity and improving the quality of seed oil are the major objectives in oil crop breeding. Natural selection has produced crops with dramatically diverse seed oil contents (SOCs) that provide the opportunity to identify the underlying mechanisms of large changes in oil accumulation through comparative biology. Soybean (*Glycine max*), cotton (*Gossypium hirsutum*), rapeseed (*Brassica napus*), and sesame (*Sesamum indicum*) are the four traditional oil crops used as sources of edible oil with sequentially increased SOCs from 20%, 30%, 40%, to 60% [1, 2], respectively. Soybean oil, the most widely consumed edible oil, is high in polyunsaturated fats [3]. Cottonseed oil contains high amounts of saturated fatty acids (FAs), which makes it more stable than other oils used for cooking [4]. Another significant oil crop is rapeseed, which is rich in unsaturated fats [5], but also has a high level of erucic acid and glucosinolates [6], which are harmful and seriously limit rapeseed as an edible oil and oil meal. Sesame with the highest oil content is considered an abundant source of oleic acid (18:1) and linoleic acid (18:2) [7]. These four oil crops have quite different seed oil contents and contain distinct FA compositions, providing attractive models for studying seed oil accumulation.

Vegetable oil is stored in the seeds of higher plants in the form of triacylglycerols (TAGs). Many genes involved in acyl-lipid metabolism (ALM) have been described in *Arabidopsis* in detail [8]. The genetic engineering of enzymes in seed oil synthesis (SOS) has successfully adjusted the level and composition of FAs such as DIACYLGLYCEROL ACYLTRANSFERASE (DGAT) [9, 10], PHOSPHOLIPID:DIACYLGLYCEROL ACYLTRANSFERASE (PDAT) [10], and ACETYL-CoA CARBOXYLASE [11, 12]. However, plant FA and lipid synthesis, desaturation, storage, and degradation pathways involve a series of complex and interconnected multienzyme system networks, and compensation mechanisms among gene family members also make mutants of certain genes have no seed oil phenotype [13, 14]. More importantly, the increase in oil accumulation that is experimentally achieved in the model plants using genetic manipulations pales in comparison with interspecies differences in SOCs. In addition, seed storage reserves mainly consist of starch, lipids, and storage proteins, and the catabolic and anabolic processes of the main storage compounds in seeds are coupled and in a dynamic equilibrium state, which is closely regulated in part by carbon partitioning at the metabolic level [15, 16]. Therefore, an in-depth study of the material distribution and accumulation in seeds is an important direction to achieve a large step forward in improving oil crops. A large-scale comparative transcriptomic analysis of oil crops that are highly diverse in their SOCs would provide more insights to identify the signatures of oil accumulation.

As next-generation high-throughput sequencing has become routine, comparative studies among different species shed new light on finding common evolutionary genes regulating the same trait at the genomic and transcriptomic levels. For example, maize *KRN2* and rice *OsKRN2* are subject to convergent selection and enhance grain yields through similar pathways [17]. The *G* gene has a conserved function in controlling seed dormancy in soybean, rice, and tomato [18]. Meanwhile, network methods are currently used to study various biological systems by exploring the relationship between observed gene products, bridging the gap from individual genes to systems biology. Systematic analyses of coexpression networks demonstrate that gene modules can be highly conserved across distant species [19].

**Fig. 1:**
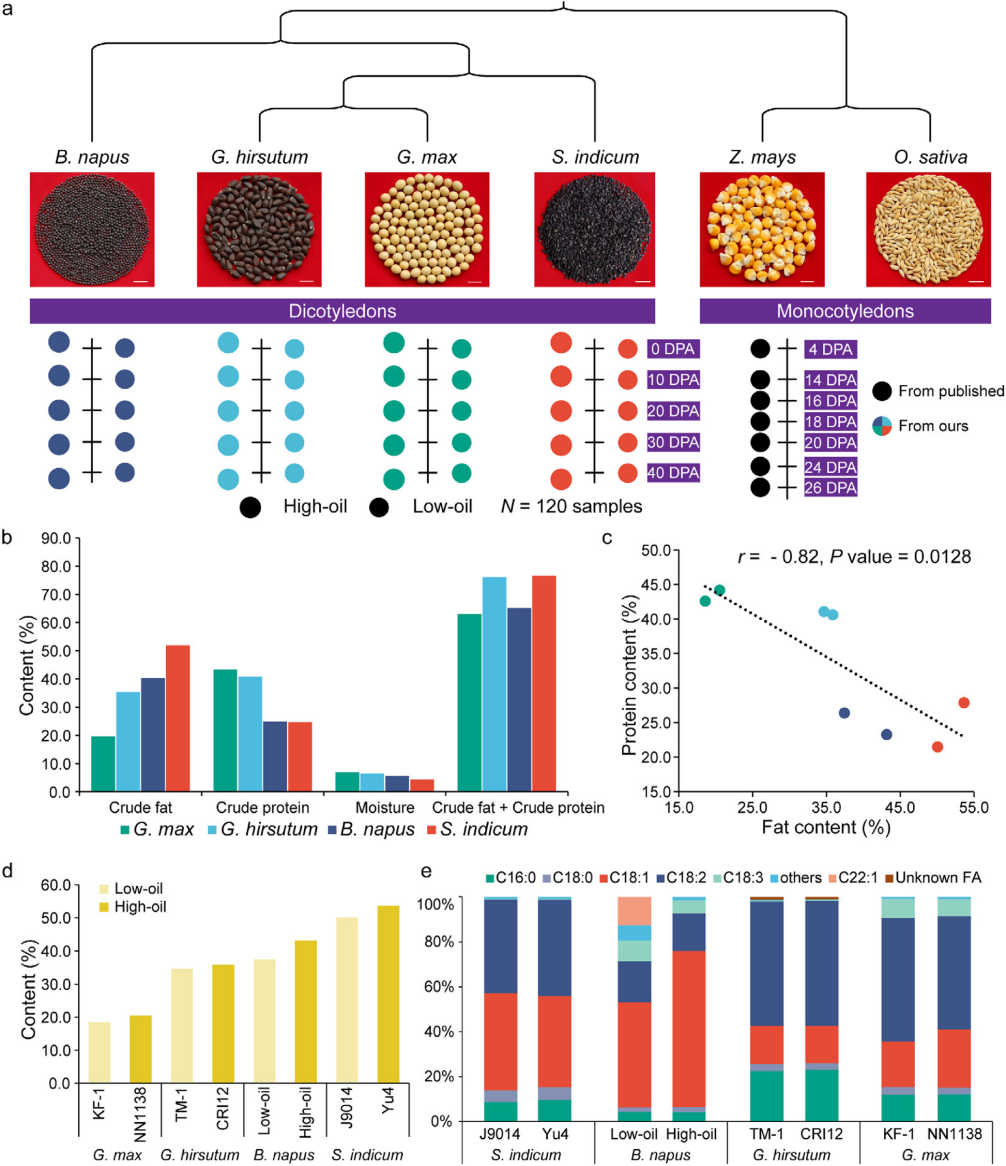
Summary of the species analyzed and seed component contents. **a** Seeds of the different species sampled for comparative transcriptomics. The samples were obtained from five different developmental stages of the high- and low-oil materials for the four oil crops. The developmental transcriptome data for maize were from the published data. **b** The crude fat, protein content, and moisture content of the mature seeds (as % seed dry weight). **c** The correlation between the fat and protein content in the oil crop seeds. Pearson’s correlation *r* =  − 0.82, *P* value = 0.0128. **d** The seed oil contents of high- and low-oil materials of the four oil crops (as % seed dry weight). **e** FA composition contents in the fully mature seeds from the four oil crops

Here, by collecting comprehensive RNA-seq resources of four oil crops with distinct SOCs, including soybean, cotton, rapeseed, and sesame (Fig. 1a), we conducted comparative transcriptomics to investigate the differences from the conserved mechanisms affecting seed oil synthesis and accumulation. Furthermore, maize (*Zea mays*) and rice (*Oryza sativa*) were also included to provide insights into the interconversion between seed storage. The findings are aimed at enriching and expanding the lipid biosynthesis metabolic mechanism, which will help breeders modify oil content and design oil crops with ideal FA compositions through genetic and metabolic engineering.

## Results

### Different SOCs and FA compositions in the four oil crops

High- and low-oil materials for each oil crop were included to generate inter- and intraspecies cross-comparisons, and the crude protein, fat content, moisture content, and FA composition of the fully mature seeds were evaluated. Different from grain crops, the carbon deposited in these oilseeds was mainly in the form of protein and fat, accounting for 63 to 77% of the total seed dry weight of each species on average (Fig. 1b and Additional file 1: Table S1). The total seed crude fat contents of soybean, cotton, rapeseed, and sesame differed from 20 to 52% (% seed dry weight), while the crude protein contents decreased from 43 to 25% successively (Fig. 1b and Additional file 1: Table S1), suggesting a significant negative correlation between the fat and protein content (Pearson’s correlation *r* =  − 0.82, *P* value = 0.0128; Fig. 1c). There were also differences in SOCs between high- and low-oil materials in each species (Fig. 1d). The detailed FA composition analysis showed that palmitic (C16:0), oleic (C18:1), and linoleic (C18:2) acids were the three major FAs in these seeds, accounting for 69 to 94%, but their composition ratios varied among the species (Fig. 1e and Additional file 1: Table S2). In soybean and cottonseed oil, linoleic acid (C18:2) (52.5% in soybean and 54.7% in cottonseed) was two- to threefold that of oleic acid (C18:1) (23.1% in soybean and 16.7% in cottonseed). Rapeseed possessed more oleic acid (C18:1) (58.0%), which was approximately threefold that of linoleic acid (C18:2) (17.4%). In sesame seed, the amount of oleic acid (C18:1) (42.0%) was almost equal to linoleic acid (C18:2) (42.1%) (Fig. 1e and Additional file 1: Table S2). In addition to the linoleic and oleic ratios, these crops had their respective FA characteristics. For example, cottonseed oil contained 26.2% saturated FAs, which was much higher than that of the other crops (14.6% in sesame, 6.6% in rapeseed, and 15.3% in soybean) (Additional file 1: Table S2). Soybean and rapeseed contained much more linolenic acid (C18:3) (8.1% and 7.5%) than cottonseed (0.4%) and sesame (0.3%), respectively (Fig. 1e and Additional file 1: Table S2). We also found that the FA profiles of the high-oil and low-oil materials in each crop were similar, except for no erucic acid (C22:1) in the high-oil rapeseed (12.6% in the low-oil material of rapeseed) (Fig. 1e and Additional file 1: Table S2). It appeared that the FA compositions were relatively stable in each species due to their genetic and physiological similarities. Overall, their remarkable divergence in SOCs and FA compositions made them excellent subjects for investigating the genetic basis of oil synthesis and accumulation.

### Higher gene numbers and expression levels shape seed oil phenotypes

**Fig. 2:**
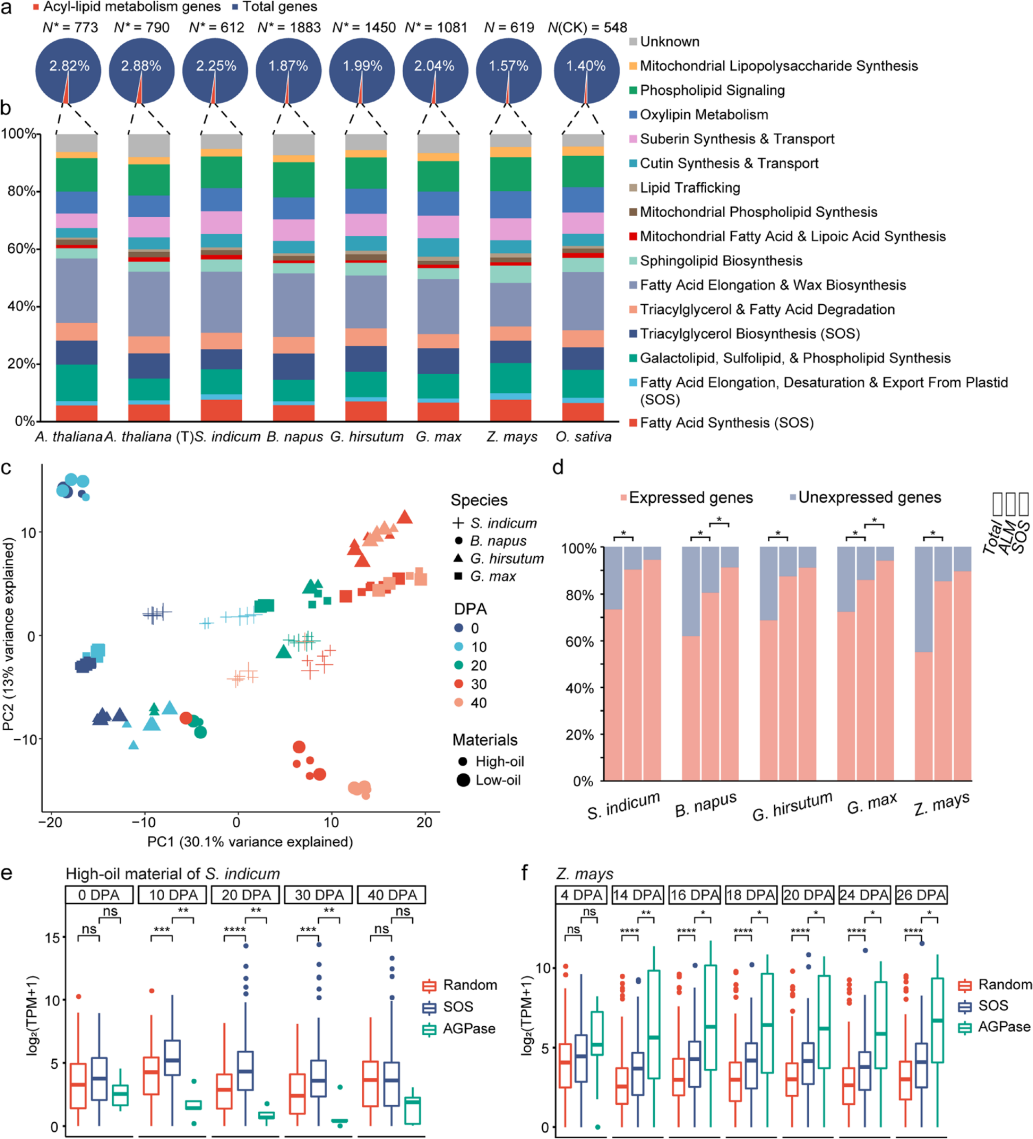
Comparison of gene numbers and transcriptomic patterns of the oil crops and maize. **a** The proportions of identified ALM genes in the total genes of each species. *N* represents the total number of ALM genes. The numbers on the pie charts represent the proportions of ALM genes to the total number of genes of each species. Comparisons of *N* were performed between the different species and rice. *P value ≤ 0.05, two-sided Fisher’s exact test. **b** Percentage of genes involved in the 16 pathways of ALM in different species. **c** PCA based on the top 500 genes with the most variable expression in each species. Each dot represents replicates of each developmental stage for each material per species. **d** The proportions of expressed genes of the total, ALM, and SOS genes in different crops. *P value ≤ 0.05, two-sided Fisher’s exact test. ALM, acyl-lipid metabolism; SOS, seed oil synthesis. **e**, **f** Comparison of expression levels between random, SOS, and AGPase genes at different developmental stages in the high-oil material of sesame and maize. ^ns^*P* value > 0.05, *P value ≤ 0.05, **P value ≤ 0.01, ***P value ≤ 0.001, ****P value ≤ 0.0001, Wilcoxon test

Number changes and differential expression in the context of gene family dynamics are driving forces of morphological and metabolic diversities in plants and play essential roles in evolutionary and developmental processes. In *Arabidopsis*, 773 genes (5 pseudogenes) identified in ALM engaged in 16 pathways for the synthesis of TAG, plastid FAs, endomembrane lipids, storage, and other functions [8] (data obtained before July 2020). By searching these ALM-related genes, 1081, 1450, 1883, 612, 619, and 548 putative genes, which were orthologous to the ALM genes of *Arabidopsis*, were identified in the *G. max*, *G. hirsutum*, *B. napus*, *S. indicum*, *Z. mays*, and *O. sativa* genomes, respectively [20–25], representing 1.40 to 2.25% of all the predicted protein-coding genes (Fig. 2a, Additional file 1: Table S3, and Additional file 2: Dataset S1). Sesame showed the highest percentage of ALM genes among the oil crops (2.25%) (Additional file 1: Table S3). *A. thaliana* (T) is a test that confirmed the validity and accuracy of the identification pipeline (see the “Methods” section). Compared to *O. sativa*, 80% of which is starch, oil crops had significantly more genes related to ALM (*P* < 0.01, two-sided Fisher’s exact test, Fig. 2a and Additional file 1: Table S3), while there was no difference between *Z. mays* and *O. sativa* (*P* = 0.05910, two-sided Fisher’s exact test, Fig. 2a and Additional file 1: Table S3). However, the proportional fractions of genes classified into 16 ALM pathways were quite similar among the seven species (Fig. 2b). Moreover, using *A. thaliana* as a dicot control, a comparison of ALM-related gene families among oil crops showed that *B. napus* had more variations in terms of the number of gene family members than the other oil crops (Additional file 3: Fig. S1). These exceptions were predominantly associated with TAG synthesis, e.g., OIL-BODY OLEOSIN (OBO) family genes (25 in *B. napus* vs eight in *A. thaliana*) and PHOSPHATIDYLCHOLINE:DIACYL-GLYCEROL CHOLINEPHOSPHOTRANSFERASE family genes (eight in *B. napus* vs two in *A. thaliana*) (Additional file 3: Fig. S1). Gene family expansion and contraction analysis also showed a high number of lipid metabolism gene families with rapid expansions in *B. napus* (branch-specific *P* value < 0.01 for the significant families (family-wide *P* value < 0.05)), including PROTEASE INHIBITOR/SEED STORAGE/LTP family (PF00234), ENOYL-ACYL CARRIER PROTEIN REDUCTASE (PF13561), MEMBRANCE BOUND O-ACYL TRANSFERASE family (PF13813), GDSL-LIKE LIPASE/ACYLHYDROLASE (PF00657), PYRUVATE KINASE (PF00224), and TYPE-2 PHOSPHATIDIC ACID PHOSPHATASE superfamily (PF01569) (Additional file 3: Fig. S1 and Additional file 4: Dataset S2). In addition, genes encoding GDSL were also significantly expanded in *G. max* with a larger variation (branch-specific *P* value = 0.000150, Additional file 3: Fig. S1 and Additional file 4: Dataset S2).

To systematically characterize transcriptomic signatures of seed oil accumulation, we collected ovules at 5 developmental stages, including 0, 10, 20, 30, and 40 days post-anthesis (DPA), from these 4 oil crops covering distinct SOC materials. A total of 4.61 billion clean reads from 120 developing seed samples were generated after filtering the reads that were low quality and too short from 5.19 billion raw reads (Additional file 5: Dataset S3). As a control, transcriptomic data for maize whole seeds at 4, 14, 16, 18, 20, 24, and 26 DPA were publicly available [26]. The average alignment rate of the oil crop samples was 85.40% (Additional file 5: Dataset S3), and RNA-seq data from maize were calculated in the same pipeline (see the “Methods” section). The Spearman’s rank correlation coefficients for the samples of each crop were all over 85% (Additional file 3: Fig. S2–S6). The first principal component (PC1), explaining 30.1% of the variance in gene expression, separated the samples by the developmental stages of seeds, and PC2 separated the samples by the various species (Fig. 2c and Additional file 3: Fig. S7). For the principal component analysis (PCA) of individual species, most of the variances (84.23 ~ 91.80%) separated the samples by developmental stages (Additional file 3: Fig. S7). For the multispecies comparative transcriptomic analysis based on coexpression networks, our analysis flow is shown in Additional file 3: Fig. S8.

Depending on the ALM genes identified in the individual species, we found that expressed genes (reached a minimum of ten reads in at least three libraries) involved in ALM showed a significantly higher proportion in each crop, highest in sesame (90.36%, Fig. 2d). Genes in “fatty acid synthesis”; “fatty acid elongation, desaturation, and export from plastid”; and “triacylglycerol biosynthesis” are directly related to seed oil synthesis and were classified as SOS genes. Their expressed ratios were much higher, especially in rapeseed and soybean (Fig. 2d). Compared with random genes in oil crops and maize, SOS genes had much higher expression levels and different durations were found in different oil crops, showing the differences in critical stages of oil accumulation (Fig. 2e and Additional file 3: Fig. S9). In contrast, genes encoding ADP-GLUCOSE PYROPHOSPHORYLASE (AGPase), a key enzyme of the starch biosynthetic pathway, were expressed at significantly higher levels than SOS genes in maize but not in oil crops (Fig. 2e, f and Additional file 3: Fig. S9), indicating interspecies differences between grain and oil crops.

### Species differences and similarities in factors affecting oil accumulation

**Fig. 3:**
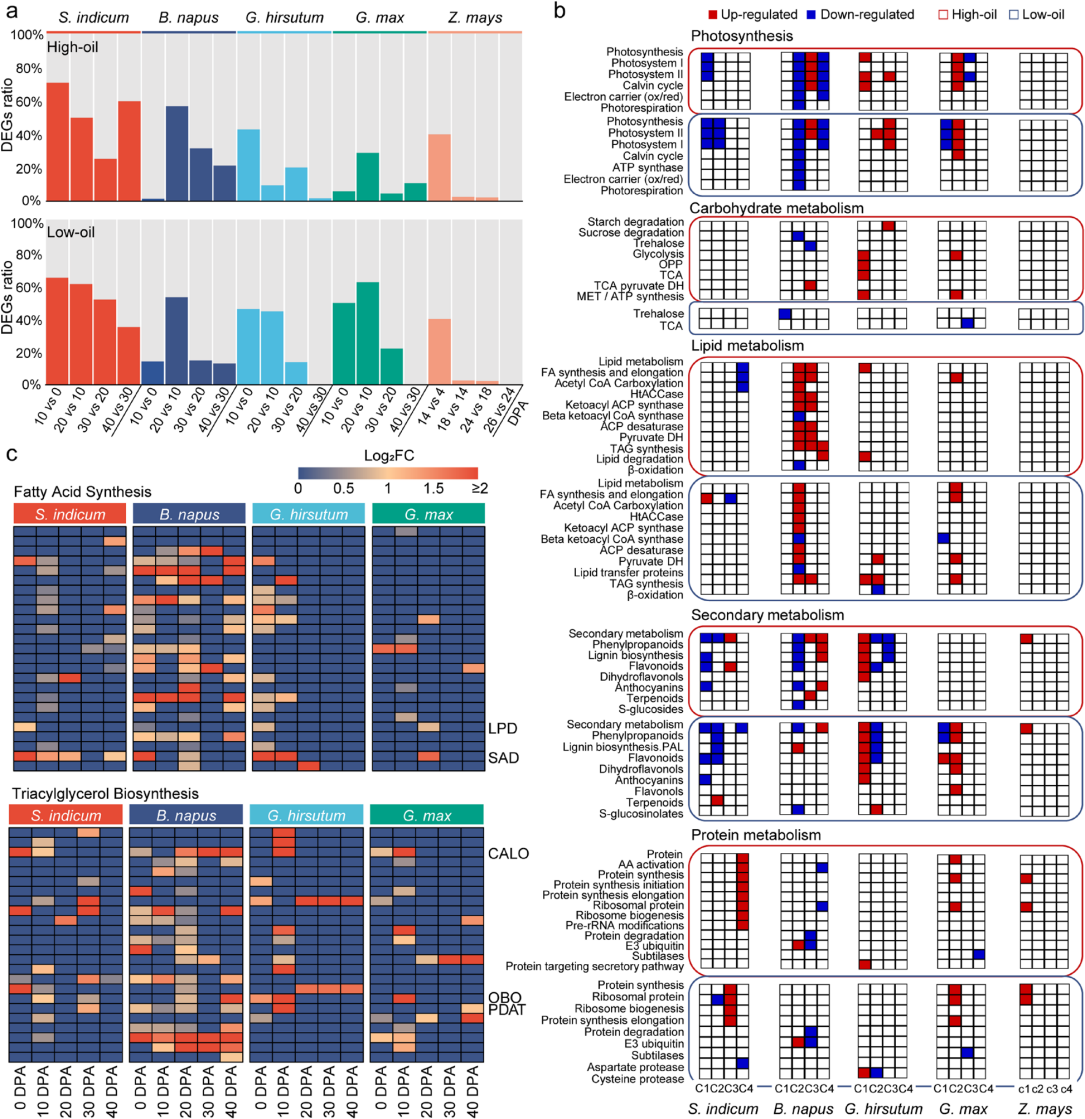
Differential expression analysis of genes in different species. **a** Number ratios of the DEGs between the adjacent developmental stages and the total expressed genes in the high-oil and low-oil materials. **b** Functional enrichment of DEGs between the adjacent developmental stages in the high-oil and low-oil materials. Red box on behalf of the upregulated DEGs in the corresponding pathways, and the blue represents those that were downregulated. Red border represents the high-oil materials, and the blue represents the low-oil materials. The C1, C2, C3, and C4 for the oil crops represent “10 vs 0 DPA,” “20 vs 10 DPA,” “30 vs 20 DPA,” and “40 vs 30 DPA,” respectively. The c1, c2, c3, and c4 for the maize represent “14 vs 4 DPA,” “18 vs 14 DPA,” “24 vs 18 DPA,” and “26 vs 24 DPA,” respectively. **c** Heatmap of the upregulated genes in “fatty acid synthesis” and “triacylglycerol biosynthesis” pathways in the high-oil materials compared to the low-oil materials in each oil crop. The median of log_2_FC of the upregulated genes represents the log_2_FC of the gene family. *Abbreviations*: LPD, dihydrolipoamide dehydrogenase; SAD, stearoyl-ACP desaturase; CALO, caleosin; OBO, oleosin; PDAT, phospholipid:diacylglycerol acyltransferase

To identify the sources of intraspecies variation in oil accumulation, we performed differential expression analysis (DEA) between high-oil and low-oil materials of each oil crop. Based on the functional enrichment of differentially expressed genes (DEGs), we compiled the categories that were critical to lipid accumulation, including photosynthesis, metabolism of carbohydrates, lipids, amino acids, secondary metabolites, and proteins. The results showed that photosynthesis, secondary, and protein metabolism formed significant differences between high- and low-oil materials (Additional file 3: Fig. S10). Between the adjacent development stages, we also conducted DEA on the samples of oil crops, as well as maize samples with the appropriate differences based on the PCA (Additional file 3: Fig. S7). The largest number of DEGs was generally observed in the comparison of “10 DPA vs 0 DPA” or “20 DPA vs 10 DPA” in the different oil crops, suggesting a dramatic change in the early stage of ovule development (Fig. 3a). Interestingly, the downregulated expression of lipid metabolism-related genes in sesame was accompanied by the upregulated expression of genes involved in protein metabolism, while in soybean, genes related to lipid metabolism and protein metabolism were simultaneously upregulated (Fig. 3b), and Gene Ontology (GO) enrichment analysis showed the same findings (Additional file 3: Fig. S11). In each oil crop, active lipid metabolism genes were always accompanied by active secondary metabolism genes that were up- or downregulated in expression (Fig. 3b). In the four oil crops, secondary and protein metabolic pathways are the two dominant metabolic pathways that have an impact on oil accumulation, but mechanisms of competition and coordinated allocation between lipid and secondary metabolism or between lipid and protein metabolism remained different.

Furthermore, DEGs in the “fatty acid synthesis” and “triacylglycerol biosynthesis” pathways between high- and low-oil materials and between different adjacent developmental stages were summarized. Only genes upregulated were considered. In the “fatty acid synthesis” of all four oil crops, genes encoding DIHYDROLIPOAMIDE DEHYDROGENASE (LPD), the E3 component of PYRUVATE DEHYDROGENASE COMPLEX (PDHC), and STEAROYL-ACYL CARRIER PROTEIN (ACP) DESATURASE were both upregulated in high-oil materials compared to low-oil materials (Fig. 3c). In the “triacylglycerol biosynthesis,” *PDAT*, *OBO*, and *CALEOSIN* were all upregulated in the high-oil materials of each oil crop (Fig. 3c). In the comparison of the adjacent developmental stages, genes in “fatty acid synthesis,” including *KETOACYL* *ACP* *SYNTHASE* I (*KAS* I), *KAS* II, *KAS* III, *KETOACYL-ACP* *REDUCTASE*, *β-PYRUVATE* *DEHYDROGENASE*, *BIOTIN CARBOXYL CARRIER PROTEIN*, *BIOTIN CARBOXYLASE*, and *α-PYRUVATE DEHYDROGENASE*, were differentially expressed between the same adjacent stages in both high- and low-oil materials of each species (Additional file 3: Fig. S12), which were mainly involved in the conversion of pyruvate to malonyl-CoA and the FA synthesis cycle. As cofactors for all reactions in FA synthesis and activators of fatty acid export from the plastid to the endoplasmic reticulum, genes encoding ACP and LONG-CHAIN ACYL-CoA SYNTHETASE were also conserved in the dynamic changes in the expression during the seed development process among species (Additional file 3: Fig. S12). Genes in the “triacylglycerol biosynthesis,” including *PDAT*, *OBO*, *FATTY ACID DESATURASE 2*, *FUSCA3*, *ABSCISIC ACID INSENSITIVE 3* (*ABI3*), *ABI4*, *CALEOSIN*, and *HIGH-LEVEL EXPRESSION OF SUGAR INDUCIBLE GENE 2/VIVIPAROUS-1/ABI3-LIKE1* (*HSI2/VAL1*), were actively expressed during development in both high- and low-oil materials of all species (Additional file 3: Fig. S13), which mainly served as transcription factors or lipid droplet proteins. A full pathway model of seed oil synthesis was summarized, and gene expression profiling showed that fatty acid synthesis (FAS) genes were expressed relatively higher and clustered more with glycolysis genes in high-oil crops, and *ABI3* and *ABI4* were expressed consistently with genes encoding oil-body proteins in sesame, cotton, and soybean but not rapeseed (Additional file 3: Fig. S14).

### The role of glycolysis in seed oil accumulation is conserved among species

**Fig. 4:**
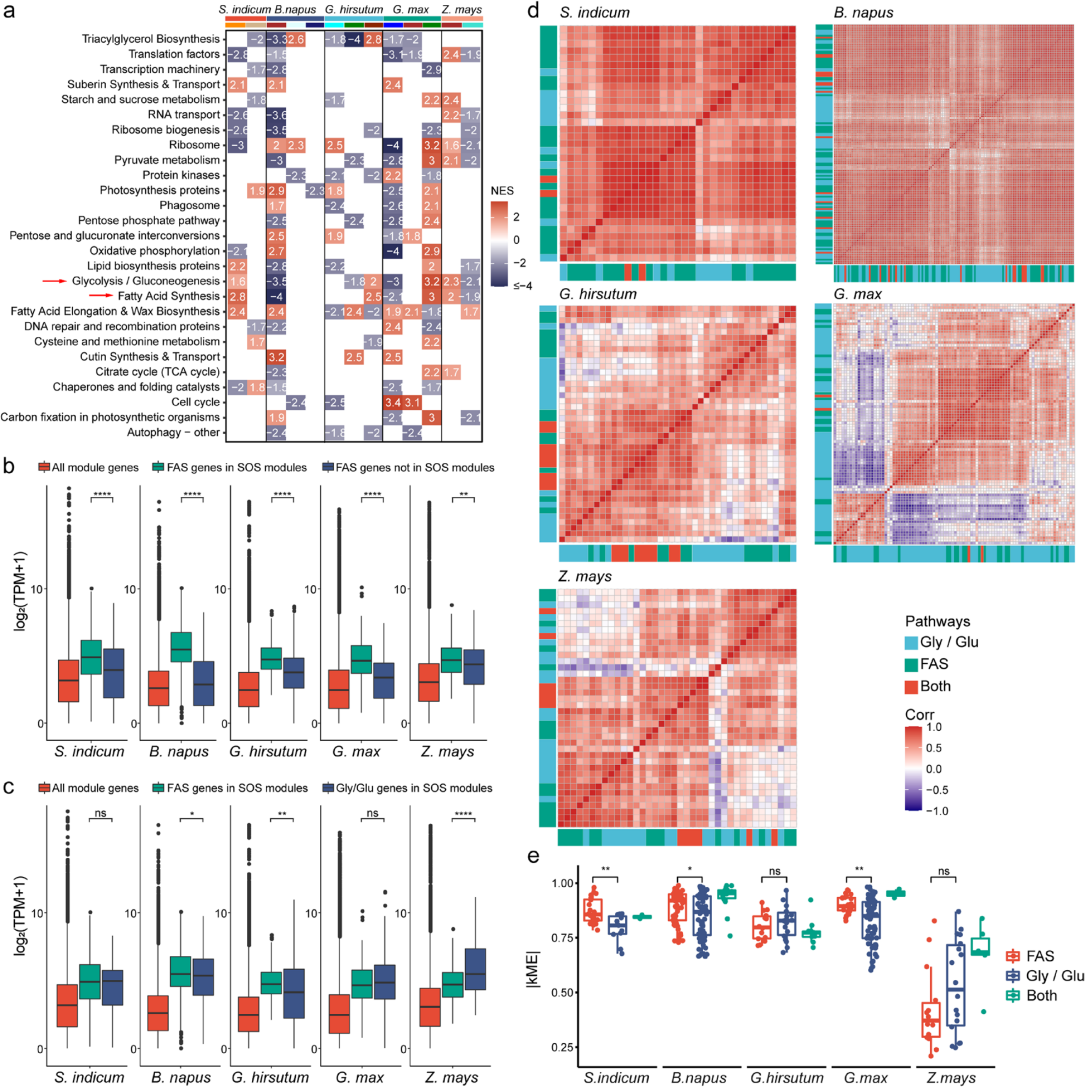
Relationships between genes in the “fatty acid synthesis” and “glycolysis/gluconeogenesis” in SOS modules in different species. **a** KEGG enrichment of SOS modules in each oil crop and maize using GSEA (categories enriched at least three times more). NES represents normalized enrichment scores which indicate the distribution of KEGG categories across a list of genes ranked by module membership kME. **b** Comparison of expression levels between FAS genes in SOS modules and not in the SOS modules in high-oil materials of each crop. ^ns^*P* value > 0.05, *P value ≤ 0.05, **P value ≤ 0.01, ***P value ≤ 0.001, ****P value ≤ 0.0001, Wilcoxon test. **c** Comparison of expression levels between “fatty acid synthesis (FAS)” genes and “glycolysis/gluconeogenesis (Gly/Glu)” genes in the SOS modules in high-oil materials of each crop. ^ns^*P* value > 0.05, *P value ≤ 0.05, **P value ≤ 0.01, ***P value ≤ 0.001, ****P value ≤ 0.0001, Wilcoxon test. **d** Heatmaps showing the Spearman correlation coefficient between FAS, Gly/Glu genes, and genes in both pathways. **e** Comparison of the absolute values of module membership kME between FAS, Gly/Glu genes, and genes in both pathways in different crops

A weighted gene coexpression network analysis was performed on the individual species to explore genes tightly linked to SOS genes. The expressed genes from sesame, rapeseed, cotton, soybean, and maize were divided into 11, 10, 9, 7, and 7 network modules, respectively (Additional file 1: Table S4 and Additional file 3: Fig. S15). Then, we identified 2 or 3 modules in each crop that were strongly associated with seed oil accumulation (Additional file 3: Fig. S16 and Additional file 6: Dataset S4, see the “Methods” section). To identify a pan-species transcriptomic signature that correlated with SOS, we ranked all the genes in the SOS modules by their Pearson correlation with the module eigengene and performed gene set enrichment analysis (GSEA). The KEGG pathways strongly enriched by the SOS modules across species included carbohydrate metabolism and amino acid metabolism (Additional file 3: Fig. S17). Interestingly, in carbohydrate metabolism, “glycolysis/gluconeogenesis” was the strongly enriched pathway common to all species (GSEA *P* < 0.05, FDR *Q* < 0.1) (Fig. 4a and Additional file 7: Dataset S5). Genes in the “fatty acid synthesis” pathway in the SOS modules were expressed significantly higher than pathway genes not in the SOS modules in each crop, ensuring strong functionality from network collaboration (Fig. 4b and Additional file 3: Fig. S18). Additionally, in the SOS modules, the expression of genes in the “fatty acid synthesis” was significantly higher than that of genes in the “glycolysis/gluconeogenesis” in rapeseed and cotton, both of which were not significantly different in sesame and soybean, while in maize, on the contrary, genes in the “glycolysis/gluconeogenesis” were expressed significantly higher than that in the “fatty acid synthesis” (Fig. 4c and Additional file 3: Fig. S18). Spearman correlation coefficients between genes expressed in “glycolysis/gluconeogenesis” and “fatty acid synthesis” in the SOS modules were calculated. We found that almost all the genes in the two pathways were positively correlated in sesame and rapeseed, while negative correlations were found in cotton, soybean, and maize (Fig. 4d). The absolute values of kME, representing the module eigengene-based connectivity, of the two pathways had a significant difference in sesame, rapeseed, and soybean, showing relatively high values of FAS genes (Fig. 4e), implying the importance of FAS genes to play hub roles in the networks.

**Fig. 5:**
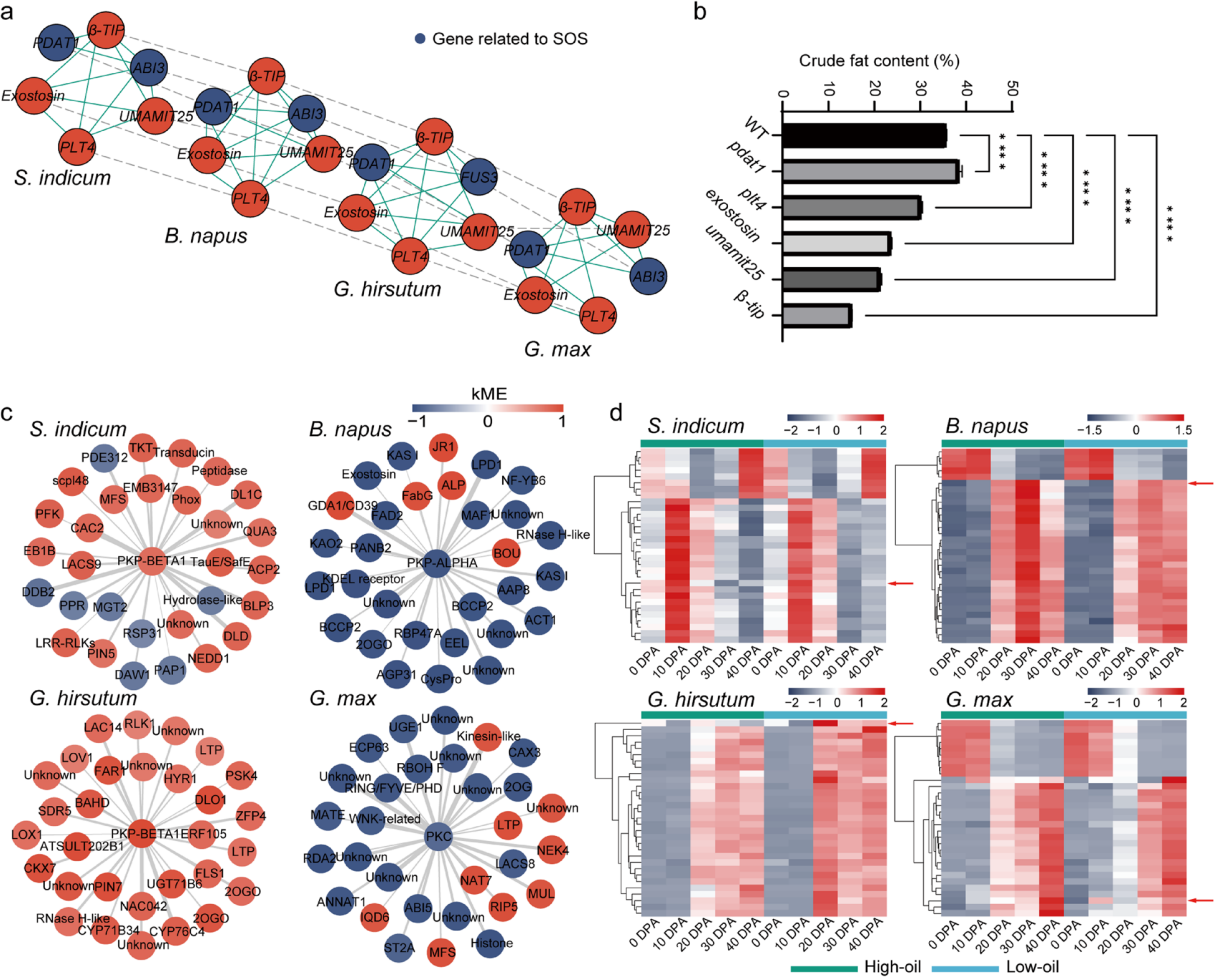
Network alignment of SOS modules across oil crops. **a** A conserved clique of the SOS coexpression networks across species. **b** Seed oil contents of the *Arabidopsis* mutants of the conserved clique single genes. ****P value ≤ 0.0001, one-way ANOVA test. **c** The top 30 genes in weights connected to the *PK*. The node color represents the kME value of the corresponding gene in the networks. **d** Heatmaps of the expression levels of the *PK* network genes of each oil crop in **c**. Red arrows point to the *PK*. The values were centered and scaled in the row direction

Network alignment is a method for efficiently integrating diverse and complex network data to identify functionally conserved networks across species. Therefore, to uncover evolutionarily conserved networks regulating oil accumulation, we merged the SOS modules in each oil crop as input, and a total of 692 aligned genes with similar network structures and sequence homology from the 4 oil crops were finally found (Additional file 8: Dataset S6). The protein domains of all the aligned network genes were reported as protein kinases, receptor tyrosine kinases, Cytochrome P450, MYBs, etc., with 465 Pfam entries common to all 4 crops (Additional file 3: Fig. S19). A conserved clique including SOS genes is shown in Fig. 5a. To determine the effects of the conserved genes on seed oil accumulation, we determined the SOCs of *Arabidopsis* mutants for 5 genes in the clique. The results showed that the SOC of the *pdat1* mutant increased by 8% compared with that of the wild type, while that of the other mutants decreased by 15 to 56% (Fig. 5b and Additional file 9: Dataset S7). Furthermore, we found genes involved in “glycolysis/gluconeogenesis” and conserved in the SOS networks of each oil crop (*SIN_1016466*, *BnaC01G0366100ZS*, *GH_D12G2756*, *Glyma.10G201100*), which encoded PYRUVATE KINASEs (PKs) catalyzing the conversion of phosphoenolpyruvate to pyruvate in glycolysis. All 4 genes had relatively high absolute values of kME, showing high intramodular connections (|kME|≥ 0.75, Additional file 10: Dataset S8). We collected the top 30 genes in weights connected to the *PKs*, and most genes were highly positively associated with the *PKs* (Fig. 5c). The expression of these genes also differed between high- and low-oil materials in each oil crop (Fig. 5d). In maize, *PK* also showed a relatively high degree of connectivity (|kME|= 0.76, Additional file 10: Dataset S8). The top 30 genes linked to *PK* in weight contained several genes related to starch metabolism, such as *STARCH SYNTHASE 2*, *STARCH BRANCHING ENZYME 2.2*, and *AGPase* (Additional file 3: Fig. S20). Among these genes, most were consistently highly expressed during the development stages (Additional file 3: Fig. S20). In summary, our cross-species screening of conserved pathway regulators provides detailed information on conserved genetic regulation of carbon source allocation and oil accumulation.

### Competing pathways of lipid metabolism are individually regulated among species

Based on the DEA, we found that secondary metabolism as well as protein metabolism affected lipid metabolism to different degrees in different oil crops; therefore, we explored and compared the relationships between genes involved in the two kinds of metabolism with SOS genes among the four species. Through the GSEA of SOS modules, “amino acid metabolism” was significantly associated with network hubs in all oil crops and maize, while “metabolism of other amino acids” was significantly associated with network hubs only in soybean (GSEA *P* < 0.05, FDR *Q* < 0.1, Additional file 3: Fig. S17 and Additional file 7: Dataset S5). Meanwhile, different types of amino acids were closely associated with the SOS network hubs among species, such as “cysteine and methionine metabolism” in sesame; “phenylalanine, tyrosine, and tryptophan biosynthesis” and “glycine, serine, and threonine metabolism” in rapeseed; “valine, leucine, and isoleucine biosynthesis” in cotton; and “alanine, aspartate, and glutamate metabolism” in soybean (Additional file 7: Dataset S5). Only “cysteine and methionine metabolism” in terms of amino acid metabolism was relatively common in the four oil crops; however, it was negatively correlated with “triacylglycerol biosynthesis” in sesame and cotton and negatively correlated with “fatty acid synthesis” in cotton but positively correlated with “fatty acid synthesis” in soybean (Fig. 4a).

**Fig. 6:**
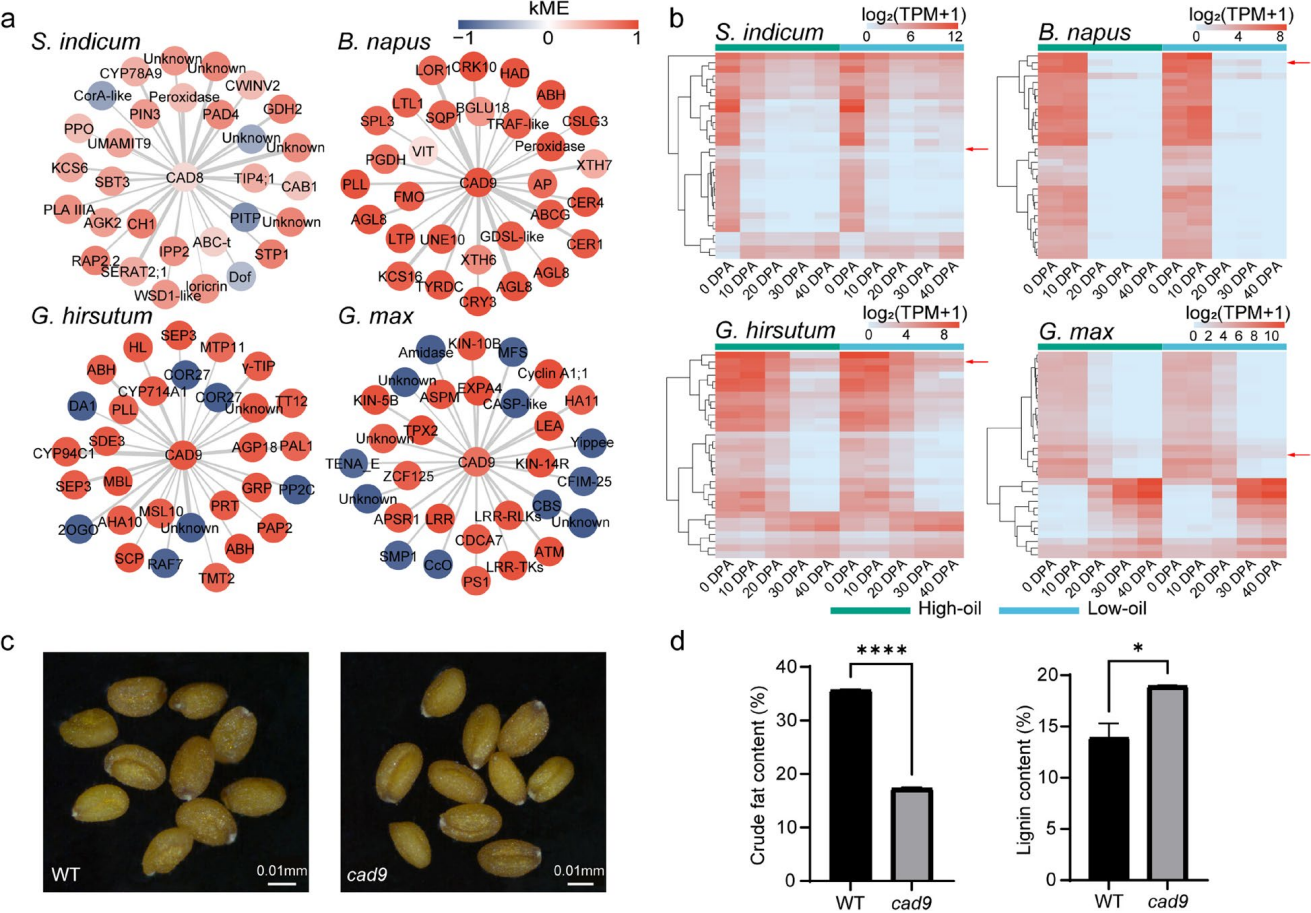
Comparison of *CAD* networks among different species and seed oil contents and lignin contents of *Arabidopsis cad9* mutants. **a** The top 30 genes in weights connected to the *CAD*. The node color represents the kME value of the corresponding gene in the networks. **b** Heatmaps of the expression levels of the *CAD* network genes of each oil crop in **a**. Red arrows point to the *CAD*. **c** Mature dry seeds of *Arabidopsis* wild type and *cad9* mutants. **d** Average seed oil contents and lignin contents of the indicated genotypes. The error bars represent the standard error of the mean, calculated from three sets of biological replicates. *P value ≤ 0.05, ****P value ≤ 0.0001, one-way ANOVA test

In addition, the “biosynthesis of other secondary metabolites” pathway in rapeseed and cotton was found to be significantly associated with network hubs (GSEA *P* < 0.05, FDR *Q* < 0.1, Additional file 3: Fig. S17 and Additional file 7: Dataset S5). Network alignment simultaneously identified the genes conserved in the SOS networks of four oil crops in “biosynthesis of other secondary metabolites” (*SIN_1005794*, *BnaC03G0683600ZS*, *GH_D03G0499*, *Glyma.14G221200*), encoding CINNAMYL ALCOHOL DEHYDROGENASE (CAD), which catalyzes the final step in a branch of phenylpropanoid synthesis specific for the production of lignin monomers [27]. When network connectivity was examined, the genes in rapeseed and cotton showed the most connections in the network with high kME values (|kME|≥ 0.90, Additional file 10: Dataset S8) but were relatively low in sesame and soybean (Fig. 6a), suggesting that physiological activities of lipid metabolism and lignin biosynthesis may be controlled through up- and downregulation of *CAD* in rapeseed and cotton. The 30 genes with the highest weights linked to *CAD* in different oil crops were also collected (Fig. 6a), and the expression level of *CAD* was relatively higher in cotton and rapeseed than that of the other 30 genes (Fig. 6b). *GhCAD9* remained highly expressed during the critical period of oil accumulation in cotton (20 DPA–30 DPA) (Fig. 6b). Similarly, we obtained *Arabidopsis* mutants of *CAD9* (Fig. 6c) and determined the crude fat and lignin content of the seeds. The *cad9* mutants showed a 52% decrease in crude fat content and a 36% increase in lignin content (Fig. 6d and Additional file 9: Dataset S7), suggesting the regulation of the relative crude fat and lignin contents by *CAD9*.

## Discussion

### Factors responsible for seed storage accumulation differences between grain and oil crops

Storage accumulation and seed structure differed greatly in monocotyledonous grain crops and dicotyledonous oil crops. In total, 70% of the maize kernel weight is starch, most of which is present in the endosperm, while maize oil is mainly confined to the germ [28]. The embryo is the major component of oil crop seeds and is the main site for lipid production and storage. In this study, when exploring the genomic factors of seed storage accumulation in different types of crops, we found that the number of ALM genes in the oil crops accounted for 2% of the total genes, which was significantly higher than that in monocotyledonous grain crops such as maize and rice (*P* < 0.01, two-sided Fisher’s exact test), suggesting the contribution of the selection of genomic variation to differences in seed storage accumulation.

Here, we examined the evolutionary processes underlying storage accumulation in different oil and grain crops from the perspective of gene coexpression relationships in a consistent framework. The results showed that genes involved in “glycolysis/gluconeogenesis” were strongly associated with the hubs of the SOS networks in both grain and oil crops and connected tightly to FAS genes, confirming that glycolysis commonly provides the production of most oil synthesis precursors [29] and suggesting that this relationship is conserved among species that accumulate different substances. More specifically, positive and negative correlations between genes in these two pathways also affect oil accumulation. Gene collaboration is essential for hierarchical regulation, which enables functional genes in the network to promote or balance each other. When more positively related genes with the same target function cooperate more closely, the function will be amplified by hubs, and the effect on the phenotype may be huge. Considering this, we can knock out or silence the hub genes that are negatively correlated or connected to the competitive pathway so as to create efficiently improved varieties. Significantly higher expression of genes in “glycolysis/gluconeogenesis” than genes in “fatty acid synthesis” was found only in maize, suggesting the presence of other “acquisitors” of glycolysis/gluconeogenesis” substrates in addition to the “fatty acid synthesis” process. However, the regulatory networks that govern the accumulation of seed storage reserves in plants are still largely unknown. Previous related studies found that TRANSPARENT TESTA GLABRA 1 plays an important role in mediating the accumulation of seed storage reserves in *Arabidopsis* [30]. In this study, we found that *PK* involved in “glycolysis/gluconeogenesis” was conserved through network alignment and highly connected in the SOS networks of each oil crop. Likewise, in maize, *PK* was highly associated with network hubs, and in particular, genes with high weights linked to *PK* included multiple genes involved in starch biosynthesis in crops [31–33]. The importance of *PK* for seed oil accumulation, grain filling, and starch synthesis has been reported for a long time; for example, a reduction in plastid PK activity in *Arabidopsis* resulted in a 60% reduction in seed oil content [34], mutation of *OsPKpα1* decreased plastid PK activity and led to a significant decrease in starch content in mutant w59 grains [35], and loss of function of *OsPK3* caused reduced PK activity and sucrose translocation defects from source to sink in rice, which led to compromised grain filling [36]. However, the conserved role of *PK* in regulating lipid accumulation in different oil crops has been little reported, and its role and interoperability in regulating the allocation of carbon sources to starch and lipids in plants are even less known.

### Controlling competitive pathways is a potential way to improve SOCs

The nonphotosynthetic plastid is an important site for the biosynthesis of starch, FAs, and nitrogen assimilation to amino acids in various plant tissues [37]. Negative correlations between protein and oil content in seeds are found in soybean [38–40], *B. juncea* [41], cottonseed [42], rapeseed [43], sesame [44], and quinoa [45]. However, positive correlations are found in maize [46] and oats [47]. For a long time, research on the key regulatory factors between oil and protein contents in seeds has been limited. From the functional enrichment of the DEGs between high- and low-oil materials of each oil crop at each development stage, we found that genes related to protein metabolism were significantly downregulated in the high-oil materials of sesame and rapeseed, which may account for the high SOC. Furthermore, although genes in the “amino acid metabolism” were significantly associated with the SOS genes in both oil crops and maize, suggesting that lipid and protein associations were prevalent in different species, the interactions were different. First, different types of amino acids were closely associated with the SOS network hubs among species, such as “cysteine and methionine metabolism” in sesame; “phenylalanine, tyrosine, and tryptophan biosynthesis” and “glycine, serine, and threonine metabolism” in rapeseed; “valine, leucine, and isoleucine biosynthesis” in cotton; and “alanine, aspartate, and glutamate metabolism” in soybean. Additionally, only “cysteine and methionine metabolism” was relatively common in the four oil crops; however, its correlations with genes involved in “triacylglycerol biosynthesis” and “fatty acid synthesis” in oil crops were different. The results revealed interspecies differences in the transformation and accumulation of proteins and oils in seeds, and it is necessary to explore key regulatory nodes based on species characteristics.

The biosynthesis of secondary metabolites was found to be strongly related to the network hubs in the single-species SOS networks of rapeseed and cotton, indicating that competition from secondary metabolites may significantly influence seed oil accumulation in rapeseed and cottonseed. Both rapeseed and cotton had shared pathways in the biosynthesis of secondary metabolites that were closely related to the hubs of the SOS networks: phenylpropanoid biosynthesis and flavonoid biosynthesis. *CAD*, which was found to be the potential regulator for lignins and lipids in SOS networks of rapeseed and cotton, catalyzes the final step in a branch of phenylpropanoid synthesis specific for the production of lignin monomers [27]. Related studies have also shown that lignin content in rapeseed coats is significantly negatively correlated with seed oil content [48, 49]. *CAD* was also functionally conserved in the SOS networks of each oil crop, and the differences in its regulatory mechanisms in different crops had critical effects on oil accumulation. A comparison of the *Arabidopsis cad9* mutant with the wild type confirmed its regulation of oil content and lignin content in seeds. The effect of *cad4* and *cad5* on the lignin contents in mature stems has been reported, whereas the induction of *cad9* did not compensate for the absence of *cad4,5* activities [50], suggesting that the emergence of functional differentiation and the regulatory role of *CAD9* still needs to be further explored.

### Regulating conserved SOS genes is an important way to improve SOCs

To date, increasing seed oil content has been successfully targeted through the elucidation and genetic modification of the oil biosynthesis pathway. However, the increase in oil accumulation that is experimentally achieved in the model organisms using genetic manipulations pales in comparison with interspecies differences in SOCs. Significantly improving different oil crops has become a key problem to be solved.

Consistent DEGs between high- and low-oil materials of different oil crops have the potential to act as key regulators to improve seed oil content. In each oil crop, genes encoding LPD were both upregulated in high-oil materials compared to low-oil materials. The dominant role of plastid PDHC in the formation of acetyl-CoA during lipid synthesis in seeds has been confirmed [51]. LPD, a member of a large family of flavoprotein oxidoreductases, completes the catalytic cycle by reoxidizing the lipoamide cofactor [52]. However, little is still known about how *LPDs* regulate lipid accumulation in plant seeds. In addition, we found that genes encoding stearoyl-ACP desaturase were differentially expressed between high- and low-oil materials of crops, which differed in FA compositions, indicating its functional conservation among species as well as its potential to engineer specialized seed oil compositions. The consistency in differential expression of *PDAT* between high- and low-oil materials among species was also found in this study, suggesting its critical role in lipid accumulation, which has been demonstrated in individual species [10, 53]. We also identified a conserved clique, including the coexpression relationship between *PDAT*, *ABI3*, and other genes among oil crops. *ABI3* was also found to be expressed consistently with genes encoding oil-body proteins, which surround discrete organelles storing oils. Thus, although research on *PDAT* has never stopped, more needs to be learned about *PDAT*’s crucial interaction with *ABI3* in the SOS network. The comparison of seed oil contents between the *Arabidopsis pdat1* mutant and wild type showed that *PDAT* had a significant increase in oil accumulation. Previous studies have shown that *PDAT1* and *DGAT1* have overlapping functions: the *dgat1-1 pdat1-2* double mutation resulted in sterile pollen that lacked visible oil bodies, and RNAi silencing of *PDAT1* in a *dgat1-1* background or *DGAT1* in a *pdat1-1* background resulted in 70 to 80% decreases in oil content [10]. This explains why the SOC increase in *pdat1* mutants may be due to the mutual compensation mechanism of *PDAT* and *DGAT*, which may be dominated by *DGAT*.

Genes with consistent differential expression changes during developmental stages were summarized, and the importance of most of them for lipid accumulation has been verified in some species in previous research. The regulatory models of ACETYL-CoA CARBOXYLASE and PDHC have been well established [54–57]. In this study, genes encoding their subunits were actively expressed during seed development. Genes involved in the FA synthesis cycle were upregulated during critical periods of FA synthesis in different crops, suggesting that the elongation of FA carbon chains is strongly conserved among species, highlighting their importance. FUSCA3, ABI3, and ABI4 are three transcription factors that were actively expressed during seed development in all species, and their positive role in lipid accumulation has been demonstrated in several species [58–64]. Furthermore, the negative regulatory effect of *HSI2/VAL1* on seed maturation has been proposed [65, 66], but its role in seed oil accumulation has not been strongly confirmed, and the role of *HSI2/VAL1* in TAG synthesis in different oil crops is presumed to be conserved from this study.

More importantly, the gene coexpression network acts as a functional amplifier and transmitter for individual genes, which cannot work independently from the community. As found in this study, the expression of FAS genes in network modules was higher than that of genes not in the network in all species. Here, we identified gene modules whose coexpression relationships were maintained across species through evolutionary history. Thus, the results move beyond the observations made in studies of individual species to provide new insights into what genes and mechanisms may be fundamental to seed oil accumulation. For example, the gathering of positive effects of gene collaboration in the “fatty acid synthesis” and “glycolysis/gluconeogenesis” pathways is crucial for oil accumulation, which is reflected not only in the correlation of gene expression in the two pathways but also in the number of function-enriched modules.

## Conclusions

Overall, we generated a dataset of seed developmental RNA-seq profiles in four species with markedly different SOCs, providing a comprehensive comparative analysis of the seed oil accumulation signatures of gene expression and collaboration. We explored the associations between the accumulation processes of the main storage compounds in seeds and found conservation and differences among grain and oil crops. Coexpression network alignment provides a way to discover key conserved genes and modules among different species. Our work moves beyond the observations made in studies of individual species to provide new insights into which genes and mechanisms may underlie seed oil accumulation from a multispecies perspective. We hope that our study can provide a valuable reference for the efficient improvement of oil crops.

## Methods

### Plant materials and growth conditions

The cotton materials from our laboratory were *G. hirsutum* acc. TM-1 (low SOC) and *G. hirsutum* cv. CRI12 (high SOC). The sesame materials J9014 (low SOC) and Yu4 (high SOC) were obtained from the Oil Crops Research Institute, Chinese Academy of Agricultural Sciences. The soybean materials KF-1 (low SOC) and NN1138 (high SOC) were obtained from the National Center for Soybean Improvement, Nanjing Agricultural University. The rapeseed materials with different SOCs came from the State Key Laboratory of Crop Genetics and Germplasm Enhancement, Nanjing Agricultural University. All the materials were grown in the field.

The control wild type was the *Arabidopsis* (*A. thaliana*) Columbia-0 accession. The T-DNA insertion lines *pdat1* (SALK_032261), *plt4* (SALK_097021), *exostosin* (SALK_018694), *umamit25* (SALK_140423), *β-tip* (SALK_125353), and *cad9* (SALK_081375) were obtained from AraShare (https://www.arashare.cn/index/). The homozygous mutant plants were identified by the three-primer (LBb1.3 + LP + RP) method (http://signal.salk.edu/tdnaprimers.2.html). We surface sterilized all seeds using 75% alcohol before sowing them on 0.8% (w/v) agar solid medium (half-strength Murashige and Skoog medium, 1% sucrose, pH 5.8–6). After breaking dormancy at 4 °C for 2 days in the dark, we allowed seeds to germinate and seedlings to grow in the culture room under 14-h light/10-h dark at 22 °C for 7 days before transplanting individual seedlings to the soil for further growth.

### Lipid and protein analysis

Total fats were extracted from frozen mature seeds, and the amounts were determined as described in Kang and Rawsthorne [67]. The contents of each FA were measured by GC/MS [68]. The total amount of FAs was calculated as the sum of all the principal components. To measure the protein content, 10 mg of seeds was homogenized in 1 mL of 50 mM 4-(2-hydroxyethyl)-1-piperazineethane-sulfonic acid/NaOH, pH 7.4, using an all-glass homogenizer.

### RNA sequencing data analysis

When these materials started flowering, the flowers were tagged and sampled randomly within a 10-day interval from 0 to 40 DPA. Three biological replicates were taken from each time point. The total RNA at five stages (0, 10, 20, 30, and 40 DPA) from different materials was sequenced with the Illumina HiSeq 2500 system using the paired-end 100-bp model. The reference genomes of soybean, cotton, rapeseed, sesame, and maize have all been released and updated (Additional file 1: Table S5) [21–25]. The clean RNA-seq reads were mapped to the reference genomes using HISAT 2.0 [69], respectively. TPM (transcripts per kilobase of exon model per million mapped reads) and the mapped read counts were calculated by StringTie (version 1.3.5) [70] and featureCounts (version 1.6.4) [71], respectively. Binary variables (total genes and genes involved in ALM and SOS were expressed or not) were compared using Fisher’s exact test [72].

The quality of the sequenced libraries was evaluated between the sample replicates using PCA (FactoMineR version 2.4) [73] and a correlation test by the Spearman method as implemented in R (version 3.6.1). The global and species-specific PCA was performed using the prcomp R package (https://www.r-project.org/) for the top 500 variation gene expression matrixes, collected from the read counts to which we applied the variance stabilizing transformation implemented in DESeq2 (version 1.12.4) [74]. The dataset libraries in which the correlation among the replicates (Spearman’s *ρ*) was lower than 0.85 were removed.

### Identification and comparison of genes involved in ALM

In total, 768 genes (after excluding 5 pseudogenes) involved in the ALM of *A. thaliana* were obtained from http://aralip.plantbiology.msu.edu/ as the query. Protein sequences of *Arabidopsis* were employed as a test for the identification process. The identified genes were required to satisfy the following three conditions: (i) Target sequences with over 33.3% identical matches to the query sequences passed Diamond (v0.9.29.130) [75] BLASTP. (ii) HMMER3.1 [76] was applied to predict the protein domains for protein sequences of the 6 crops, with significance thresholds defined by “-E 1e-5” and “–domE 2e-20.” Gene pairs with the same domains were chosen. (iii) The default parameters (− *I* = 1.5) for OrthoFinder (version 2.4.0) [77] were used to assign the genes for each crop and *Arabidopsis* to a unique orthogroup. Binary variables (genes involved in ALM or not with a certain species and rice) were compared using a “two-sided” Fisher’s exact test [72].

### Gene family expansion and contraction analysis

To understand the evolutionary changes of the lipid metabolism-related gene families among these four oil crops, gene family expansion and contraction analysis were performed for the four crops, maize, rice, and *Arabidopsis*. First, orthologs and paralogs for all seven species were collected from the previous identification from OrthoFinder (version 2.4.0) [77] and then used to construct a STAG species evolutionary tree. The predicted divergence years of rice and *Arabidopsis* were collected as a standard from Timetree (http://www.timetree.org/) for r8s (v. 1.81) to construct an over-metric tree, adjusting the phylogenetic tree’s scale to time. Then, CAFÉ (Version 4.2.1) [78] was used to determine the significantly evolved orthogroups after excluding the gene families with a notable copy number variation (existing only in one species and *N* > 100). To determine the significance of the expansion and contraction of the orthogroups, the *P* values were calculated for each orthogroup based on a Monte Carlo resampling procedure, and the threshold for a significant expansion and contraction was set to a *P* value < 0.05 (family-wide *P* value). The branch-specific *P* values were obtained by the Viterbi method with a randomly generated likelihood distribution. This method calculates the exact *P* values for transitions between parent and child family sizes for all branches of the phylogenetic tree. A low branch-specific *P* value (< 0.01) indicates a rapidly evolving branch. Finally, the annotations of the orthogroups were performed according to the Pfam database (http://pfam.xfam.org/) using HMMER [76].

### Differential gene expression analysis and functional enrichment analysis

The differential gene expression analysis was conducted in R (version 3.6.1) using the package DESeq2 (version 1.12.4) [74]. The count tables from featureCounts (version 1.6.4) [71] were used as input, and only genes that reached a minimum of 10 reads in at least three libraries were retained. The selection of *P* values was controlled for a false discovery rate (*Q* value) by the BH method [79] at *α* = 0.05. After constructing a unified standard genome-wide functional annotation for the five species through Mercator (https://www.plabipd.de/portal/web/guest/mercator-sequence-annotation), Mapman [80] was used to perform functional enrichment analysis of DEGs for each species. The model was ORA-FISHER, and Benjamini Yekutilie was used for multiple testing corrections. GO enrichment analysis of DEGs was performed with the ClusterProfiler (v3.14.0) [81] package in R (version 3.6.1).

### Weighted gene coexpression network analysis

The weighted gene coexpression network analysis (WGCNA) package [82] in R (version 3.6.1) was used to build the single-species weighted gene coexpression networks. The rlog normalized output of the merged expressed gene matrix from the high-oil and low-oil materials from DESeq2 (version 1.12.4) [74] was used as the input for WGCNA. The adjacency matrix was constructed from the matrix of Pearson correlations between all pairs of genes across the samples using an appropriate soft threshold for each crop. Network modules were defined using a dynamic tree-cutting algorithm [83], with a minimum module size of 100. Genes belonging to different modules were allocated to different colors, with genes not assigned the color gray. The module eigengene was calculated as the first principal component of the standardized module expression profiles. The module membership of a gene, kME, representing intramodular connectivity was estimated by the Pearson correlation between the expression level of that gene and the module eigengene [84].

### Identification of seed oil synthesis modules and gene set enrichment analysis

The identification of SOS modules was performed by two methods [85]. Genes involved in FA synthesis, FA elongation, desaturation and export from plastid, and TAG biosynthesis were selected as SOS genes. First, SOS genes in each oil crop and maize were enriched in the network modules using the “greater” Fisher’s exact test [72]. Thus, the modules with more SOS genes were chosen (*P* value < 0.05). The second method used a GSEA [86] to select modules in which SOS genes played important hub roles (GSEA *P* < 0.05, FDR *Q* < 0.1). Sets of genes related to SOS and genes ranked by kME were subjected to the GSEA function in the R package ClusterProfiler (v3.14.0) [81] to compare the organization of SOS coexpression networks in different species. The functional enrichment of the SOS network hubs in each species (SOS module networks merged) was performed using GSEA on gene sets derived from Kyoto Encyclopedia of Genes and Genomes (KEGG) databases with a significance threshold of *P* < 0.05 and FDR *Q* < 0.1.

### Network alignment analysis

A global alignment of multiple gene coexpression networks across species was performed using *NetCoffee* (version 1.0) [87]. *NetCoffee* searches for a global alignment by maximizing a target function using simulated annealing. The network files needed by *Netcoffee* came from the merged coexpression network of the SOS modules and were filtered by a threshold of weight > 0.1. The sequence similarity efiles of each pair of species were obtained from Diamond (version 0.9.29) [75] BLASTP sequence alignment results. The parameter representing how much topology contributes to the alignment score was set to 0.5 (− alpha = 0.5). Cytoscape v3.9.0 [88] was used for network visualizations. The Gasoline [89] plugin of Cytoscape v3.9.0 was used for the local alignment of the conservative networks from *NetCoffee*. The BLAST *E* value was also needed for similarity information. All the parameter settings were default (the density threshold was 0.8; sigma, representing the minimum node degree of the initially aligned proteins, was 3).

### Supplementary Information

**Additional file 1: Table S1.** Main components of mature seeds. **Table S2.** FA profiles of seeds from the materials in different crops. **Table S3.** Gene counts of every ALM pathway in different crops. **Table S4.** Parameters and properties of weighted gene coexpression networks of individual species. **Table S5.** Summary of reference genomes.

**Additional file 2: Dataset S1.** ALM-related genes identified in different species.

**Additional file 3:** **Fig. S1.** Comparison of different crops in gene family size. **Fig. S2–S6.** Spearman’s rank correlation of sample transcript expression profiles of the four oil crops and maize. **Fig. S7.** PCA of sample transcript expression profiles. **Fig. S8.** Analysis flow of multispecies comparative transcriptomic analysis based on coexpression networks. **Fig. S9.** Comparison of the expression levels of random, SOS, and AGPase genes at different developmental stages in different materials. **Fig. S10.** Ratios and functional enrichment of the DEGs between the high-oil and low-oil materials. **Fig. S11.** GO terms on biological processes shared between high- and low-oil materials of different species for DEGs between the adjacent developmental stages. **Fig. S12.** DEGs in ‘Fatty Acid Synthesis’ that were upregulated in the latter developmental stages compared to the former in each crop. **Fig. S13.** DEGs in ‘Triacylglycerol Biosynthesis’ that were upregulated in the latter developmental stages compared to the former in each crop. **Fig. S14.** A full pathway model of oil accumulation in plant seeds and expression profiling of these genes in the ovules of the four oil crops. **Fig. S15.** Hierarchical clustering trees showing coexpression modules identified using WGCNA. **Fig. S16.** Hierarchical clustering dendrograms of the eigengenes of each module in each oil crop. **Fig. S17.** Venn diagram of KEGG ko2 pathways associated with the hubs of SOS modules in each oil crop. **Fig. S18.** Comparison of expression levels between pathway genes in the low-oil materials. **Fig. S19.** Venn diagram of the protein domain annotations of 692 aligned genes in conserved networks and the top 20 PFAM annotations in counts in each species. **Fig. S20.** The *PK* network in maize and its expression profile.

**Additional file 4: Dataset S2.** Gene family expansion and contraction analysis between oil crops, rice, maize, and *Arabidopsis*.

**Additional file 5: Dataset S3.** Summary of RNA-seq libraries.

**Additional file 6: Dataset S4.** Identified modules related to seed oil synthesis.

**Additional file 7: Dataset S5.** GSEA on KEGG pathways of SOS modules in different species.

**Additional file 8: Dataset S6.** Conserved network genes among the four oil crops.

**Additional file 9: Dataset S7.** Crude fat, protein, and lignin contents of *Arabidopsis* mutants and wild type seeds.

**Additional file 10: Dataset S8.** Key genes in the KEGG pathways enriched in the SOS modules.

## Data Availability

All cotton materials are available from the corresponding author. The raw RNA sequencing data of all four oil crops generated in this study is free for download from the National Center for Biotechnology Information (NCBI) SRA using project code PRJNA862748 (https://www.ncbi.nlm.nih.gov/bioproject/PRJNA862748) [90]. The raw RNA sequencing data of maize, used in the manuscript, is available in the NCBI SRA under accession number SRP037559 (https://www.ncbi.nlm.nih.gov/sra/?term=SRP037559) [91].

## Abbreviations

SOC: Seed oil content
*G. max*: *Glycine max*
*G. hirsutum*: *Gossypium hirsutum*
*B. napus*: *Brassica napus*
*S. indicum*: *Sesamum indicum*
*Z. mays*: *Zea mays*
*O. sativa*: *Oryza sativa*
*A. thaliana*: *Arabidopsis thaliana*
KEGG: Kyoto Encyclopedia of Genes and Genomes
GO: Gene Ontology
FA: Fatty acid
TAG: Triacylglycerol
ALM: Acyl-lipid metabolism
SOS: Seed oil synthesis
DGAT: Diacylglycerol acyltransferase
PDAT: Phospholipid:diacylglycerol acyltransferase
DPA: Days post-anthesis
TPM: Transcripts per kilobase of exon model per million mapped reads
PCA: Principal component analysis
DEG: Differentially expressed gene
WGCNA: Weighted gene coexpression network analysis
GSEA: Gene set enrichment analysis
DEA: Differential expression analysis
OBO: Oil body oleosin
LPD: Dihydrolipoamide dehydrogenase
PDHC: Pyruvate dehydrogenase complex
ACP: Acyl carrier protein
KAS: Ketoacyl-ACP synthase
ABI3: Abscisic acid insensitive 3
HSI2/VAL1: Sugar inducible gene2/viviparous-1/ABI3-like1
FAS: Fatty acid synthesis
PK: Pyruvate kinase
CAD: Cinnamyl alcohol dehydrogenase
AGPase: ADP-glucose pyrophosphorylase
