# Supplementary material for: Comparative transcriptomic analysis provides insights into the genetic networks regulating oil differential production in oil crops
Source: BMC Biol. 2024 May 13;22:110. doi: 10.1186/s12915-024-01909-x (PMC11089805; doi:10.1186/s12915-024-01909-x)
Supplement: Supplementary file 1 — Additional file 1: Table S1. Main components of mature seeds. Table S2. FA profiles of seeds from the materials in different crops. Table S3. Gene counts of every ALM pathway in different crops. Table S4. Parameters and properties of weighted gene coexpression networks of individual species. Table S5. Summary of reference genomes. [file 12915_2024_1909_MOESM1_ESM.pdf]

Table S1 Main components of mature seeds (as % seed dry weight)

Content (%)	*S. indicum*			*B. napus*			*G. hirsutum*			*G. max*		
	Low J9014	High Yu4	Mean	Low Low-oil	High High-oil	Mean	Low TM-1	High CRI12	Mean	Low KF-1	High NN1138	Mean
Fat	50.08	53.66	51.870	37.44	43.19	40.315	34.68	35.87	35.275	18.59	20.54	19.565
Protein	21.48	27.88	24.680	26.40	23.25	24.825	41.06	40.58	40.820	42.58	44.16	43.370
Fat + Protein	71.56	81.54	76.550	63.84	66.44	65.140	75.74	76.45	76.095	61.17	64.70	62.935
Moisture	4.44	4.29	4.365	6.11	5.02	5.565	7.21	5.76	6.485	6.89	6.72	6.805

Table S2 FA profiles of seeds from the materials in different crops (%)

FA composition	*S. indicum*			*B. napus*			*G. hirsutum*			*G. max*		
	Low J9014	High Yu4	Mean	Low Low-oil	High High-oil	Mean	Low TM-1	High CRI12	Mean	Low KF-1	High NN1138	Mean
C14:0	0.00	0.00	0.000	0.06	0.06	0.060	0.71	0.68	0.695	0.08	0.08	0.080
C16:0	8.74	9.59	9.165	4.34	4.11	4.225	22.29	22.85	22.570	12.01	12.07	12.040
C16:1	0.14	0.15	0.145	0.27	0.27	0.270	0.59	0.52	0.555	0.11	0.11	0.110
C18:0	5.10	5.70	5.400	1.93	2.41	2.170	2.98	2.80	2.890	3.35	2.94	3.145
C18:1	43.31	40.59	41.950	46.92	69.16	58.040	16.78	16.55	16.665	20.27	25.94	23.105
C18:2	41.45	42.66	42.055	18.09	16.64	17.365	54.54	54.94	54.740	54.82	50.27	52.545
C18:3	0.32	0.30	0.310	9.20	5.89	7.545	0.53	0.35	0.440	8.55	7.65	8.100
C20:0	0.60	0.67	0.635	0.69	0.47	0.580	0.28	0.25	0.265	0.29	0.32	0.305
C20:1	0.20	0.19	0.195	4.98	0.81	2.895	0.00	0.00	0.000	0.17	0.20	0.185
C20:2	0.000	0.000	0.000	0.24	0.00	0.120	0.00	0.00	0.000	0.00	0.00	0.000
C22:0	0.14	0.15	0.145	0.40	0.17	0.285	0.00	0.00	0.000	0.35	0.42	0.385
C22:1	0.00	0.00	0.000	12.60	0.00	6.300	0.00	0.00	0.000	0.00	0.00	0.000
Unknown FA	0.00	0.00	0.000	0.00	0.00	0.000	1.30	1.07	1.185	0.00	0.00	0.000
C24:1	0.00	0.00	0.000	0.45	0.00	0.225	0.00	0.00	0.000	0.00	0.00	0.000

Table S3 Gene counts of every acyl-lipid metabolism pathway in different crops

Pathway	*A. thaliana*	*A. thaliana* (T)	*S. indicum*	*B. napus*	*G. hirsutum*	*G. max*	*Z. mays*	*O. sativa*
Fatty Acid Synthesis	52	56	57	130	120	87	57	43
Fatty Acid Elongation, Desaturation & Export From Plastid	14	14	14	29	26	19	17	12
Galactolipid, Sulfolipid, & Phospholipid Synthesis	117	71	65	169	153	111	78	64
Triacylglycerol Biosynthesis	77	83	52	205	154	117	58	52
Triacylglycerol & Fatty Acid Degradation	57	55	43	130	105	64	37	39
Fatty Acid Elongation & Wax Biosynthesis	206	212	158	495	316	250	113	134
Sphingolipid Biosynthesis	33	33	32	81	76	50	45	33
Mitochondrial Fatty Acid & Lipoic Acid Synthesis	10	14	12	20	15	15	9	11
Mitochondrial Phospholipid Synthesis	17	18	12	37	36	17	12	10
Lipid Trafficking	7	8	7	20	22	19	11	6
Cutin Synthesis & Transport	30	39	35	96	88	85	34	28
Suberin Synthesis & Transport	47	67	59	169	133	102	57	49
Oxylipin Metabolism	70	70	60	171	149	109	70	58
Phospholipid Signaling	107	102	82	275	188	138	88	72
Mitochondrial Lipopolysaccharide Synthesis	20	24	20	56	44	37	27	22
Unknown	57	75	38	164	95	86	33	28
Unigene	773	790	612	1883	1450	1081	619	548
Genome predict genes	27416	27416	27148	100919	72761	52872	39498	39045
Fisher's *P* value (compare to O.sativa)	< 2.2e-16	< 2.2e-16	5.17E-16	1.43E-09	5.49E-13	1.95E-13	0.05910	1
percentage	2.82%	2.88%	2.25%	1.87%	1.99%	2.04%	1.57%	1.40%

Table S4 Parameters and properties of weighted gene coexpression networks of individual species

Dataset	Network size	Power (β)	Scale free topology model fit (signed R^2)	Mean connectivity	Slope	Module ID	Module size
Cotton (high-oil and low-oil)	46969	15	0.809	364	-1.630	cyan, green, greenyellow, grey, lightgreen, lightyellow, orange, orangered4, red (9)	4908, 8629, 9315, 10163, 3012, 3057, 1107, 4747, 2031
Rapeseed (high-oil and low-oil)	57165	16	0.913	528	-1.100	black, brown, green, grey, grey60, lightcyan, lightgreen, magenta, midnightblue, purple (10)	3001, 24175, 2878, 13619, 1594, 4010, 834, 2202, 3508, 1344
Sesame (high-oil and low-oil)	18929	14	0.582	343	-1.200	bisque4, brown, brown4, darkgreen, darkgrey, darkorange, floralwhite, green, grey, lavenderblush3, tan (11)	2858, 1602, 998, 3477, 288, 1796, 981, 3839, 854, 136, 2100
Soybean (high-oil and low-oil)	34703	14	0.728	527	-1.550	black, blue, brown, green, greenyellow, grey, tan (7)	2580, 15091, 5202, 2297, 491, 7274, 1768
maize	21666	20	0.800	68	-2.060	black, blue, brown, grey, pink, turquoise, yellow (7)	608, 5194, 1640, 5402, 2179, 5056, 1587

Table S5 Summary of reference genomes

**Species**	**Cultivar**	**Genome size**	**Release year**	**Source**
G. hirsutum	TM-1	2295Mb	2019	http://cotton.zju.edu.cn/
B. napus	ZS11	921.5Mb	2021	http://cbi.hzau.edu.cn/bnapus/
S. indicum	zhongzhi13	259.7Mb	2016	/
G. max	Wm82	978Mb	2019	http://www.phytozome.net
Z. mays	B73	2106Mb	2017	https://www.maizegdb.org/
O. sativa	Nipponbare	373Mb	2013	http://rice.plantbiology.msu.edu/
