# Supplementary material for: Comparative transcriptomic analysis provides insights into the genetic networks regulating oil differential production in oil crops
Source: BMC Biol. 2024 May 13;22:110. doi: 10.1186/s12915-024-01909-x (PMC11089805; doi:10.1186/s12915-024-01909-x)
Supplement: Supplementary file 3 — Additional file 3: Fig. S1. Comparison of different crops in gene family size. Fig. S2–S6. Spearman’s rank correlation of sample transcript expression profiles of the four oil crops and maize. Fig. S7. PCA of sample transcript expression profiles. Fig. S8. Analysis flow of multispecies comparative transcriptomic analysis based on coexpression networks. Fig. S9. Comparison of the expression levels of random, SOS, and AGPase genes at different developmental stages in different materials. Fig. S10. Ratios and functional enrichment of the DEGs between the high-oil and low-oil materials. Fig. S11. GO terms on biological processes shared between high- and low-oil materials of different species for DEGs between the adjacent developmental stages. Fig. S12. DEGs in ‘Fatty Acid Synthesis’ that were upregulated in the latter developmental stages compared to the former in each crop. Fig. S13. DEGs in ‘Triacylglycerol Biosynthesis’ that were upregulated in the latter developmental stages compared to the former in each crop. Fig. S14. A full pathway model of oil accumulation in plant seeds and expression profiling of these genes in the ovules of the four oil crops. Fig. S15. Hierarchical clustering trees showing coexpression modules identified using WGCNA. Fig. S16. Hierarchical clustering dendrograms of the eigengenes of each module in each oil crop. Fig. S17. Venn diagram of KEGG ko2 pathways associated with the hubs of SOS modules in each oil crop. Fig. S18. Comparison of expression levels between pathway genes in the low-oil materials. Fig. S19. Venn diagram of the protein domain annotations of 692 aligned genes in conserved networks and the top 20 PFAM annotations in counts in each species. Fig. S20. The PK network in maize and its expression profile. [file 12915_2024_1909_MOESM3_ESM.pdf]

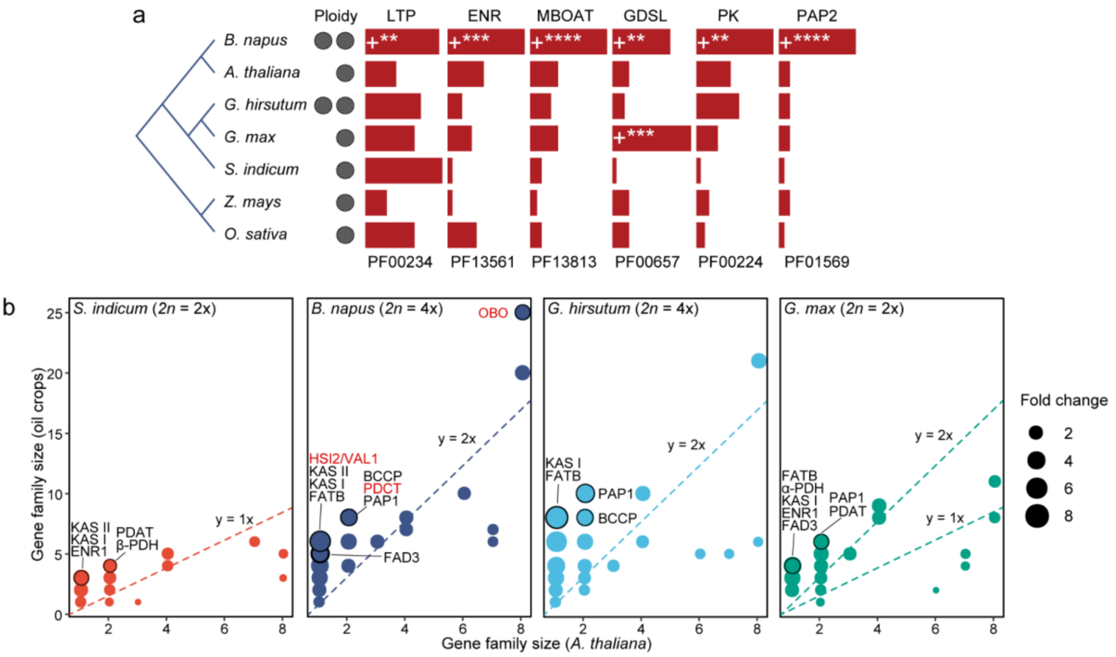

Figure S1 Comparison of different crops in gene family size. (a) Expansions in the lipid-related gene families among 7 species. For ploidy, one, two circles represent diploid, tetraploid, respectively. + indicates an increase in gene family members relative to the corresponding parent family. * *P* value ≤ 0.05; ** *P* value ≤ 0.01; *** *P* value ≤0.001; **** *P* value ≤ 0.0001. The identification of branches for which the evolution was significantly rapid using the Viterbi method with the randomly generated likelihood distribution. LTP, protease inhibitor/seed storage/LTP family; ENR, enoyl-acyl carrier protein reductase; MBOAT, membrane bound O-acyl transferase family; GDSL, GDSL-like lipase/acylhydrolase; PK, pyruvate kinase; PAP2, type 2 phosphatidic acid phosphatase superfamily. (b) Dot matrix plot of the fold changes in the gene family sizes of the ALM genes identified in the four oil crops compared with *A. thaliana*. KAS II, 3-ketoacyl-ACP synthase II; KAS I, 3-ketoacyl-ACP synthase I; ENR1, enoyl-ACP reductase 1; PDAT, phospholipid:diacylglycerol acyltransferase; β-PDH, β-pyruvate dehydrogenase; α-PDH, α-pyruvate dehydrogenase; HSI2/VAL1, HIGH-LEVEL EXPRESSION OF SUGAR INDUCIBLE GENE2/VP1/ABI3-LIKE1; FATB, fatty acyl thioesterase B; BCCP, biotin carboxyl carrier protein, subunit of heteromeric acetyl-CoA carboxylase; PDCT, phosphatidylcholine:diacylglycerol cholinephosphotransferase; PAP1, phosphatidic acid phosphatase 1; FAD3, fatty acid desaturase 3.

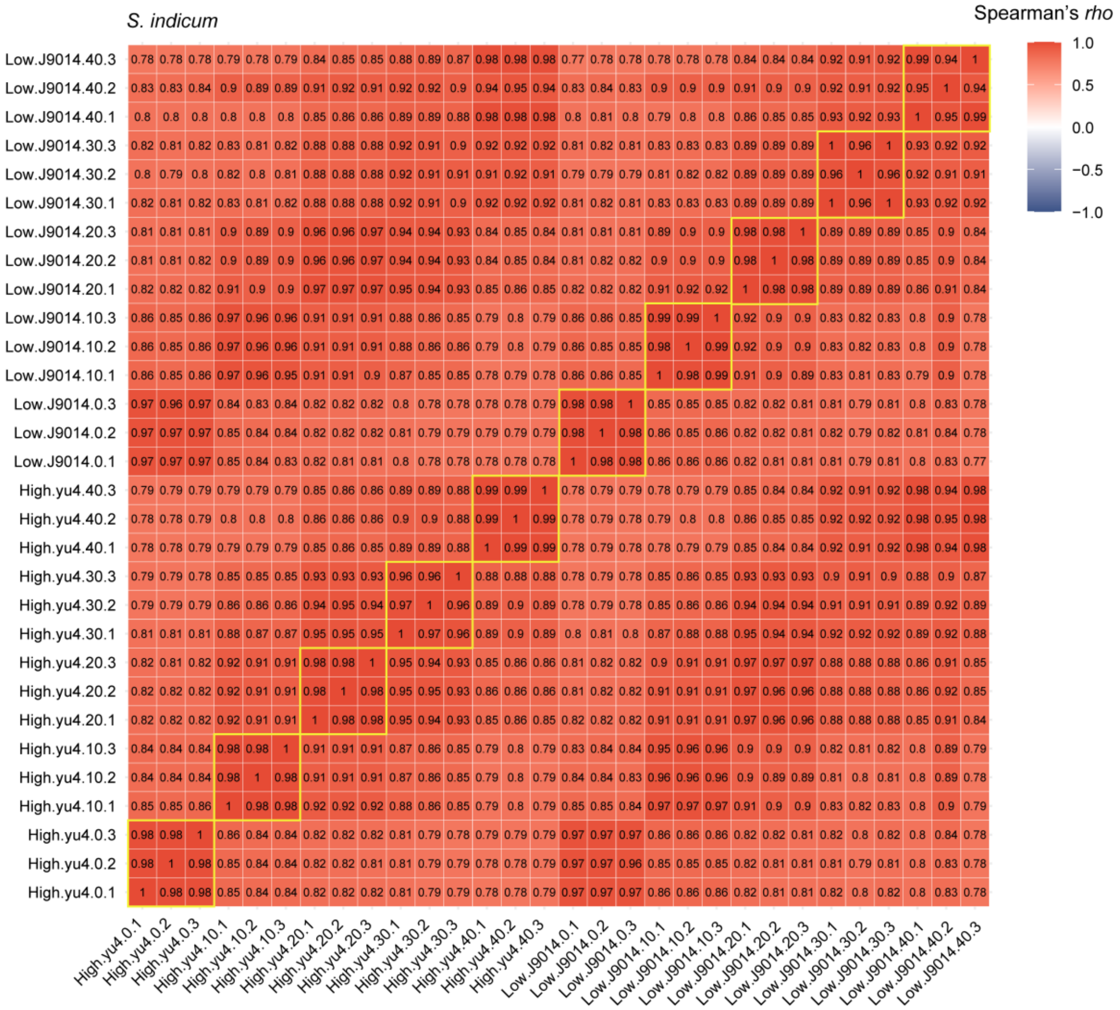

Figure S2 Spearman's rank correlation of sample transcript expression profiles of *S. indicum*.

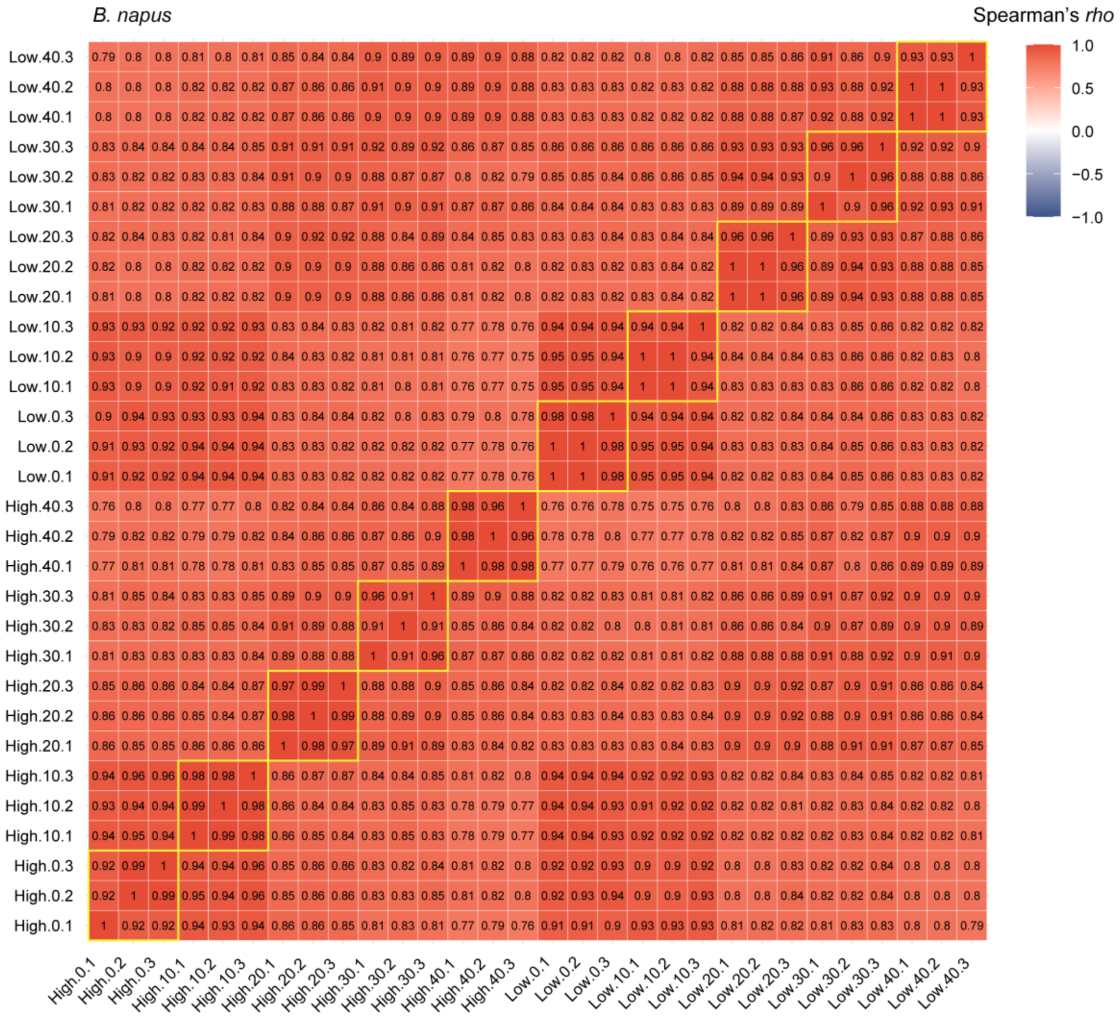

Figure S3 Spearman's rank correlation of sample transcript expression profiles of *B. napus*.

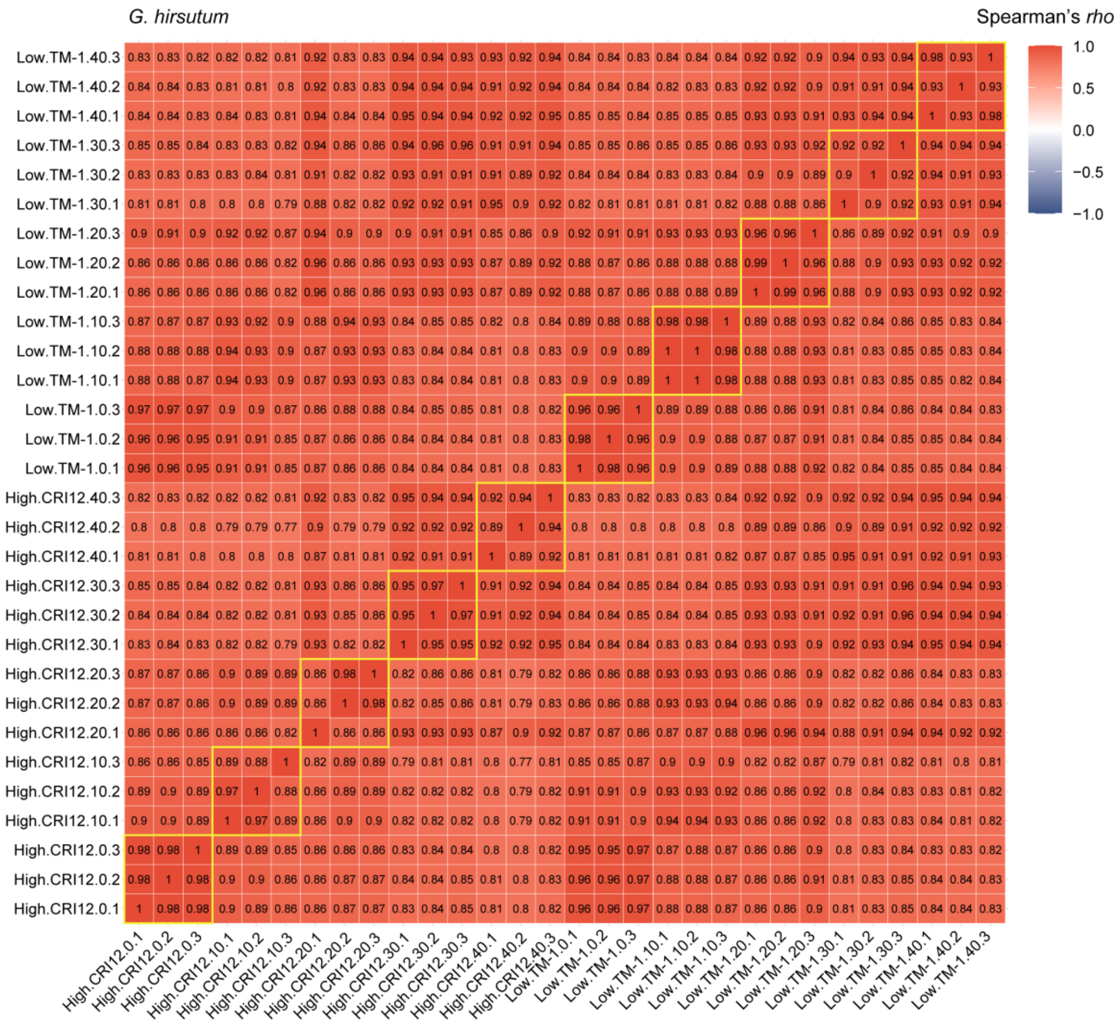

Figure S4 Spearman's rank correlation of sample transcript expression profiles of *G. hirsutum*.

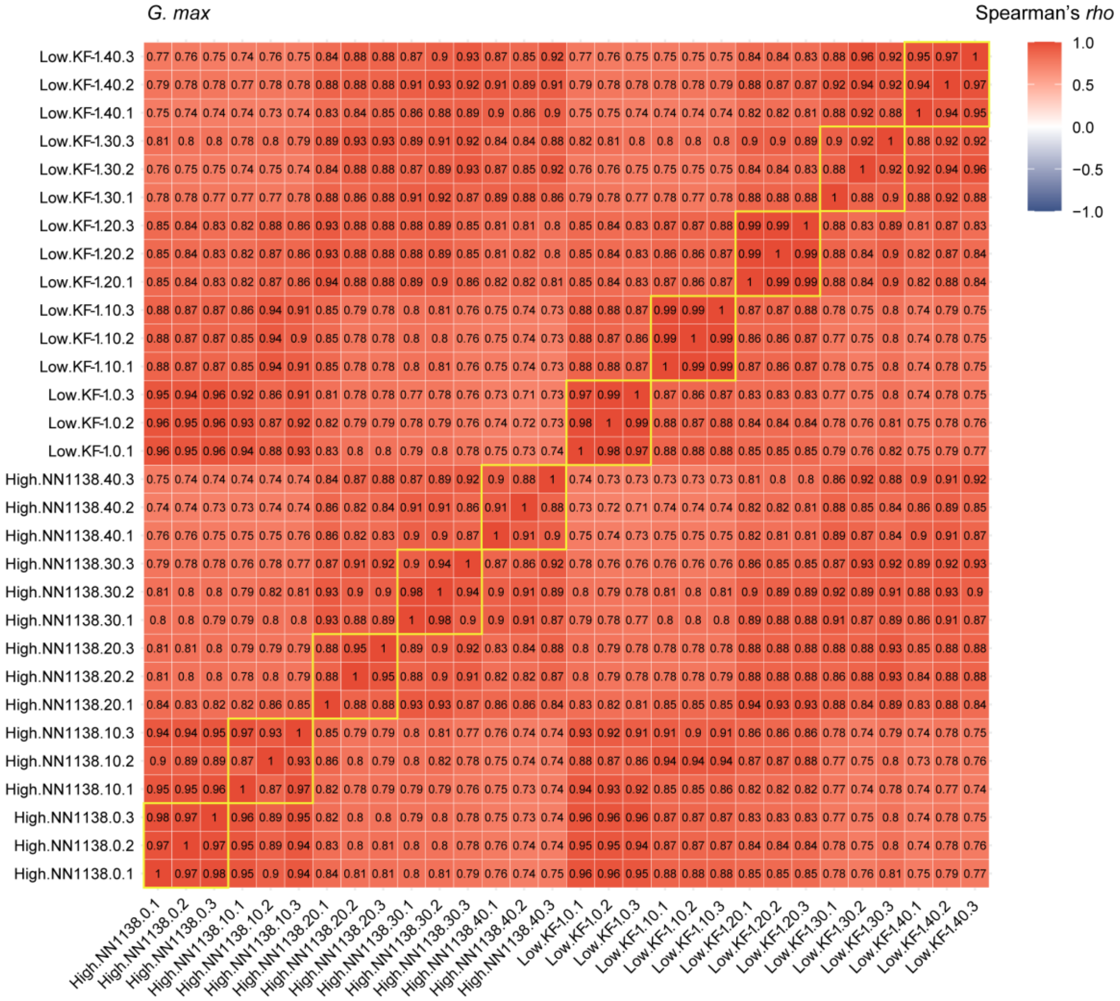

Figure S5 Spearman's rank correlation of sample transcript expression profiles of *G. max*.

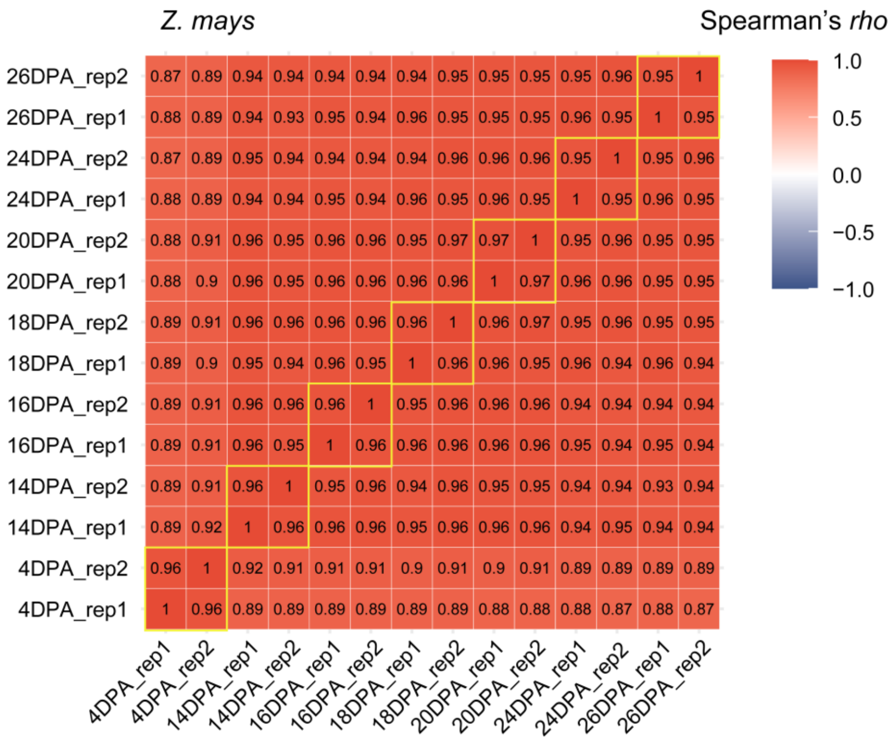

Figure S6 Spearman's rank correlation of sample transcript expression profiles of *Z. mays*.

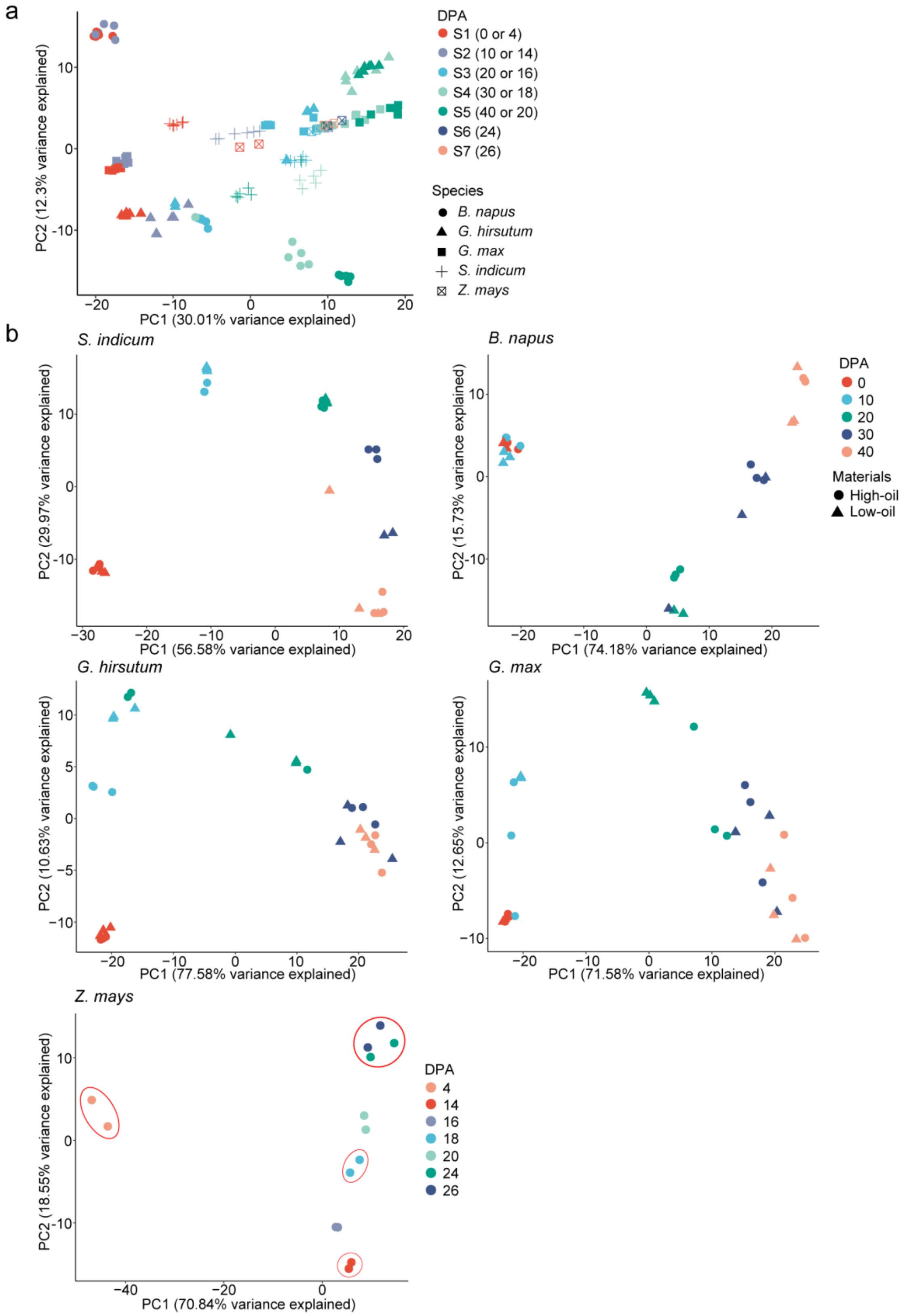

Figure S7 Principal component analysis (PCA) of sample transcript expression profiles. (a) The PCA of the oil crop and maize samples. (b) The PCAs of the individual species.

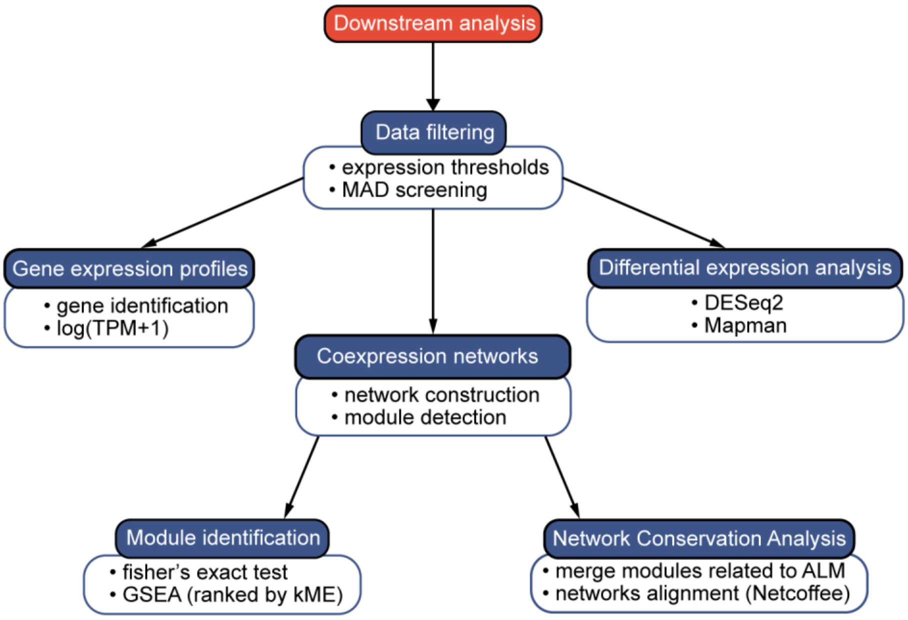

Figure S8 Analysis flow of multispecies comparative transcriptomic analysis based on coexpression networks.

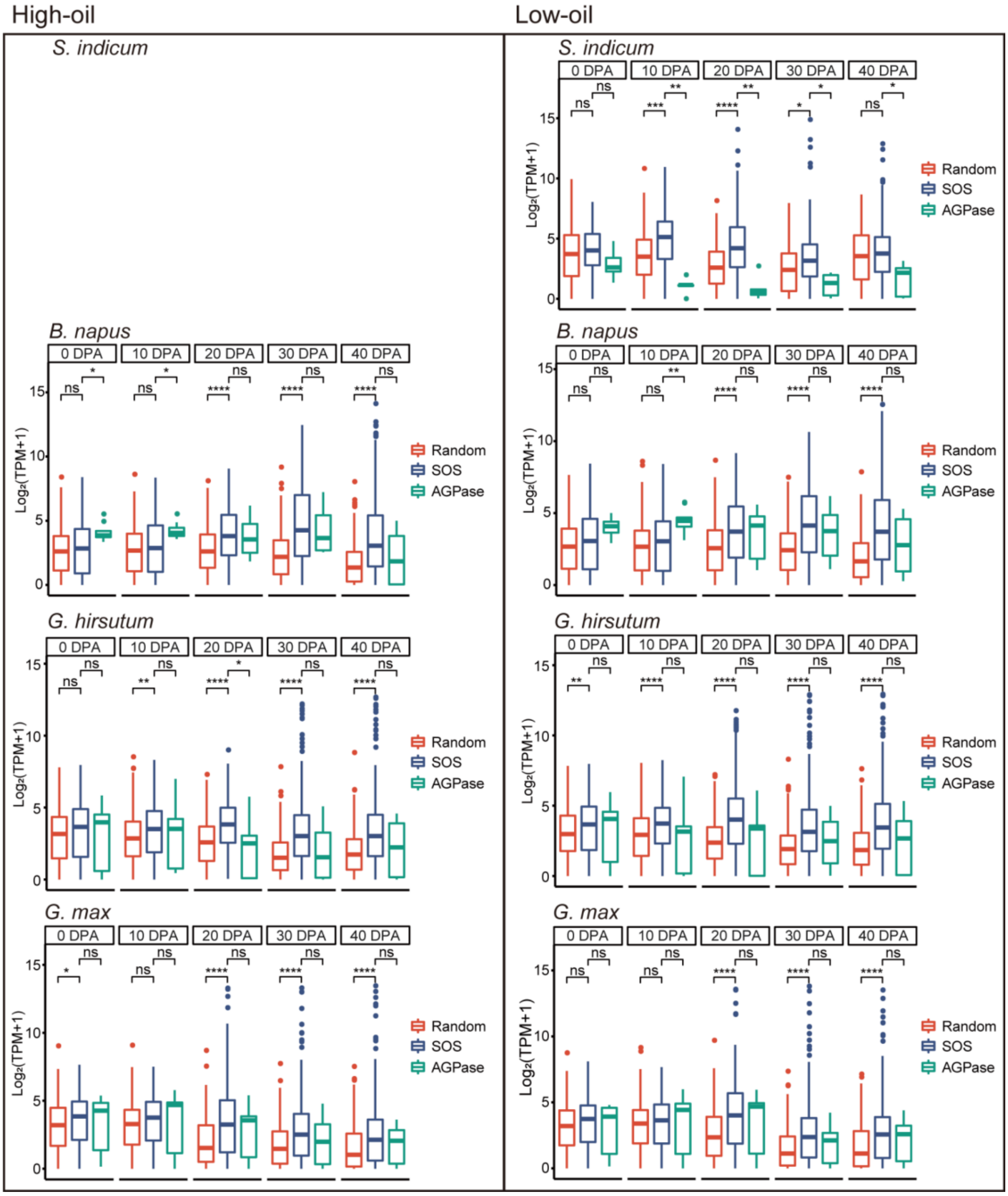

Figure S9 Comparison of the expression levels of random, SOS, and AGPase genes at different developmental stages in different materials. ns *P* value > 0.05; * *P* value ⩽ 0.05; ** *P* value ⩽ 0.01; *** *P* value ⩽ 0.001; **** *P* value ⩽ 0.0001, Wilcox test.

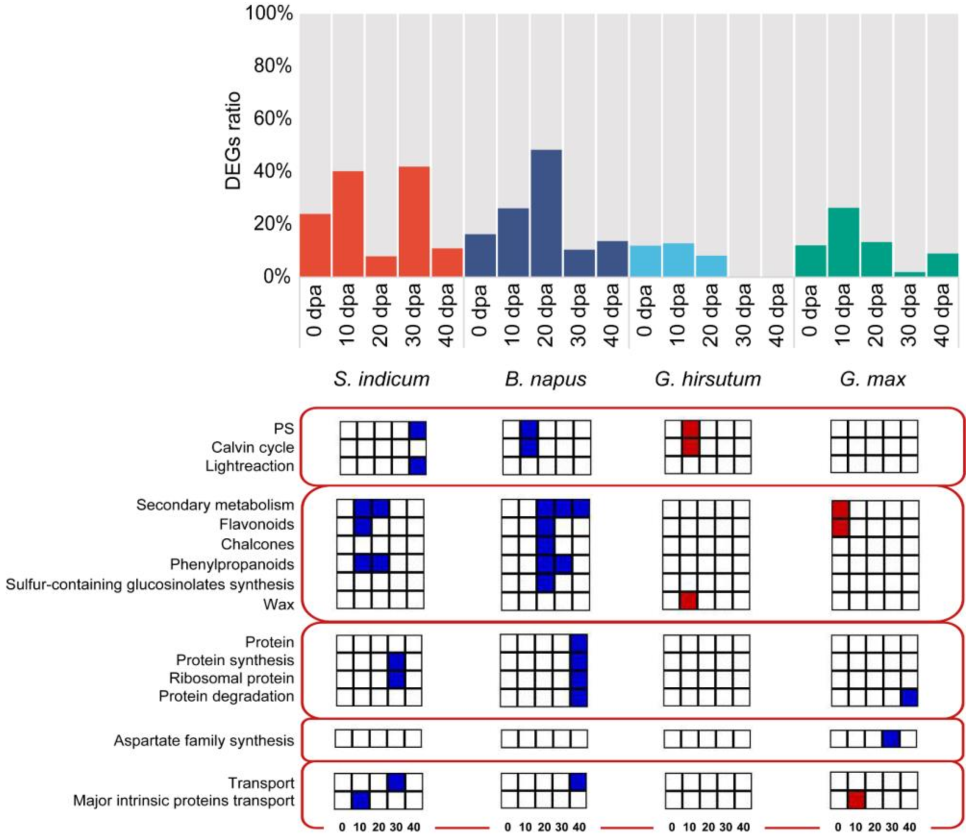

Figure S10 Ratios and functional enrichments of the DEGs between the high-oil and low-oil materials. Red box on behalf of the up-regulated DEGs in the high-oil materials were enriched in the corresponding pathways, and the blue represents down-regulated DEGs.

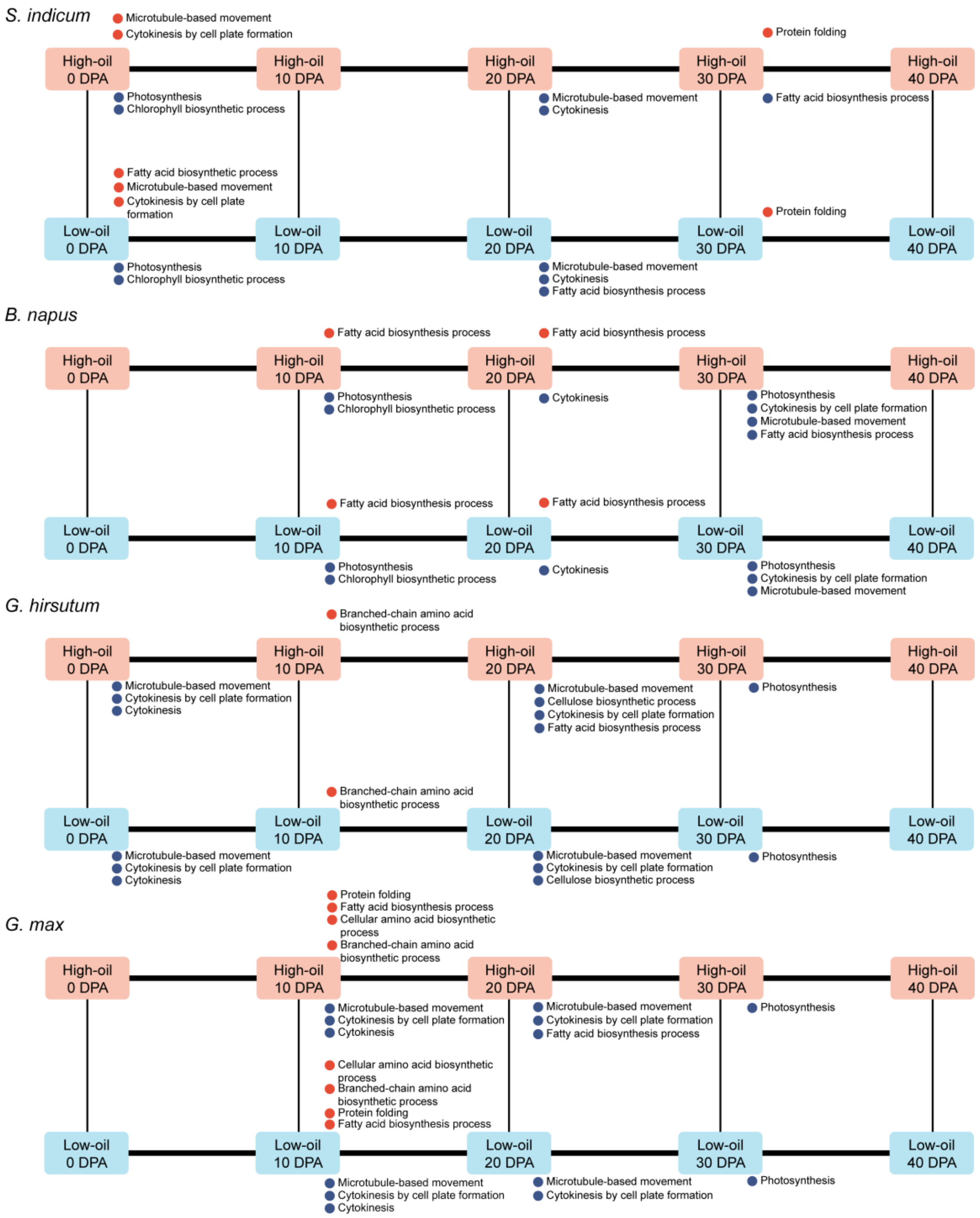

Figure S11 GO terms on biological processes shared between high- and low-oil materials of different species for DEGs between the adjacent developmental stages. Red circles indicated enrichment of up-regulated genes, blue indicated downregulated.

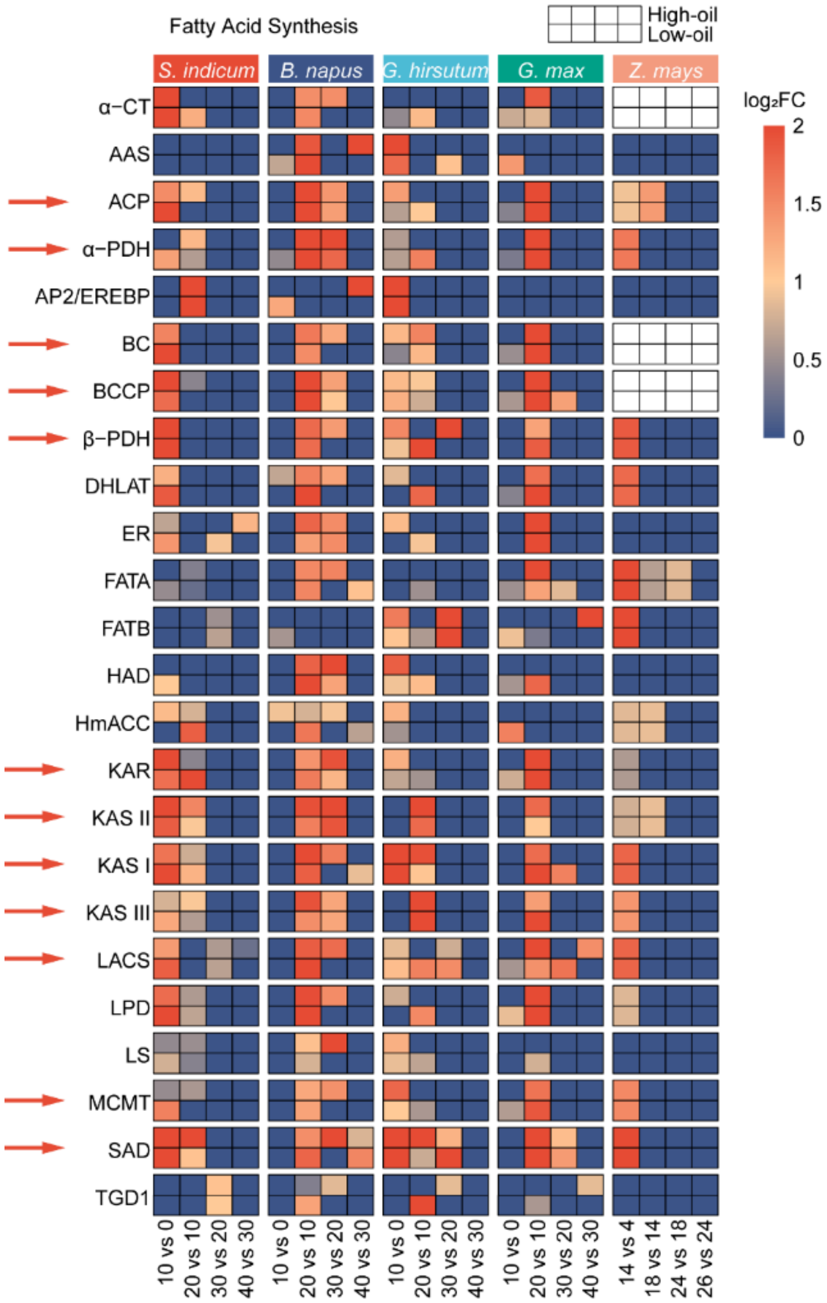

Figure S12 DEGs in 'Fatty Acid Synthesis' that were up-regulated in the latter developmental stages compared to the former in each crop. Blank boxes indicated that the gene family was absent in the species. The median of log_2FC of the up-regulated genes represents the log_2FC of the gene family.

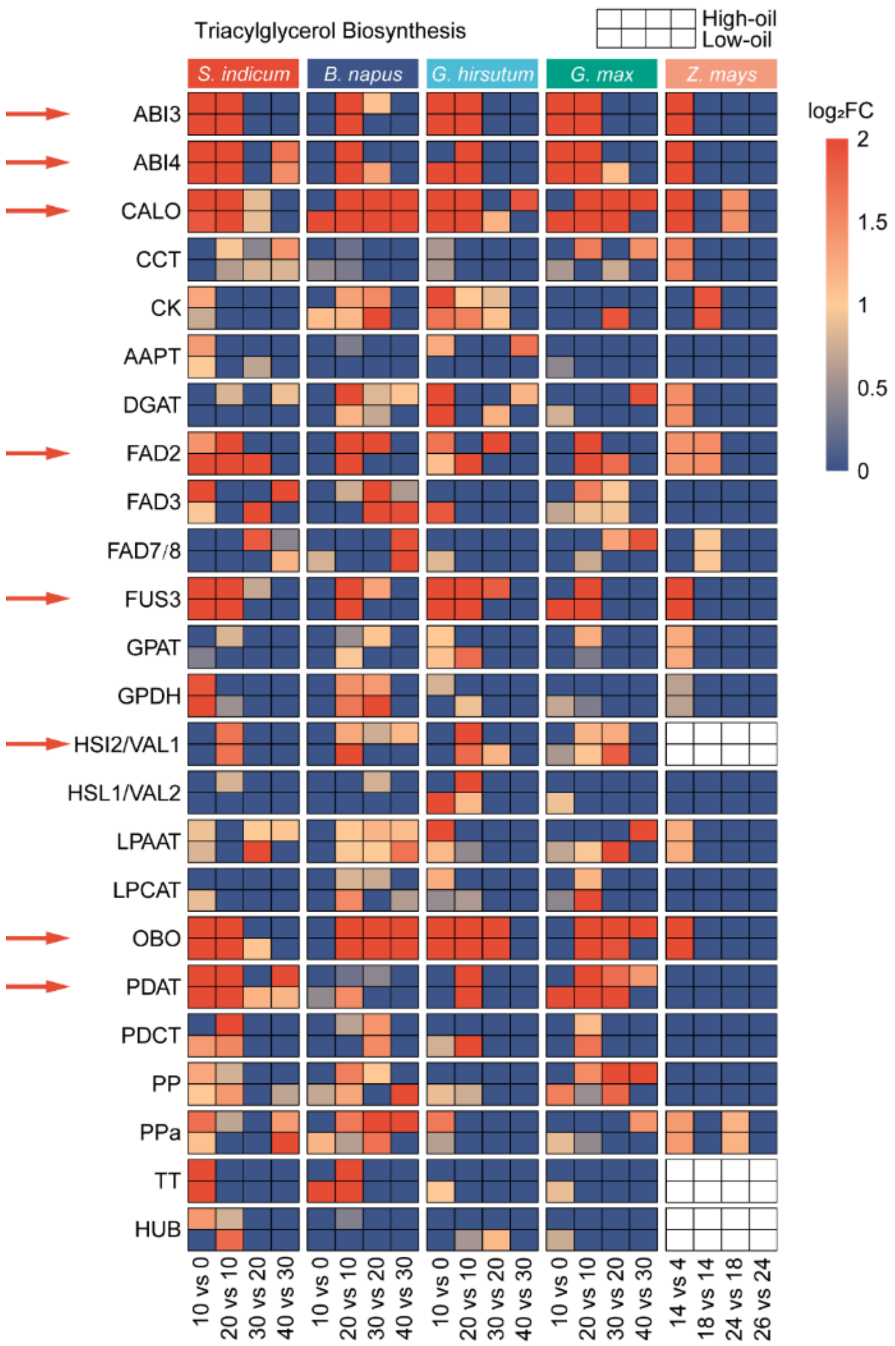

Figure S13 DEGs in 'Triacylglycerol Biosynthesis' that were up-regulated in the latter developmental stages compared to the former in each crop. Blank boxes indicated that the gene family was absent in the species. The median of log_2FC of the up-regulated genes represents the log_2FC of the gene family.

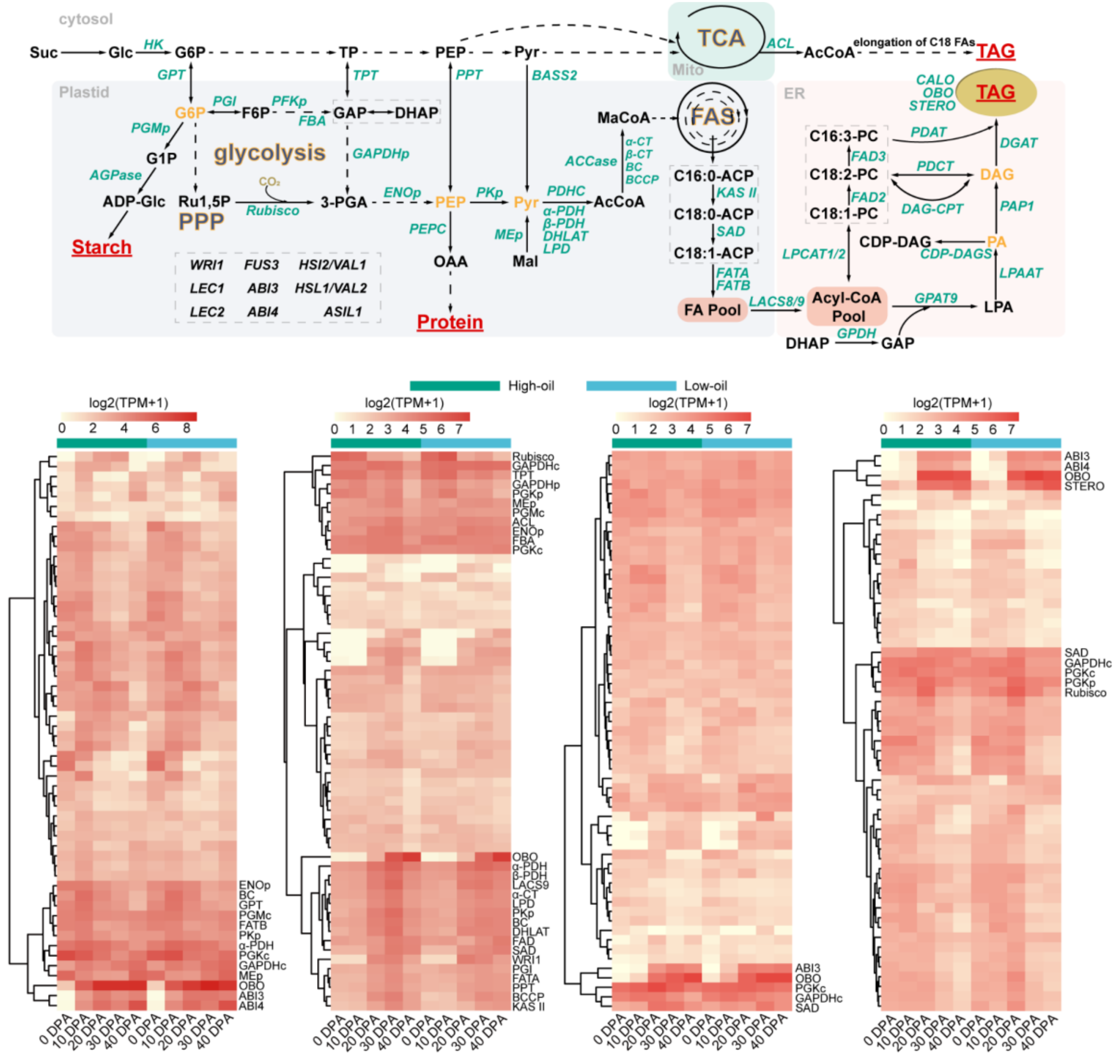

Figure S14 A full pathway model of oil accumulation in plant seeds and expression profiling of these genes in the ovules of the four oil crops.

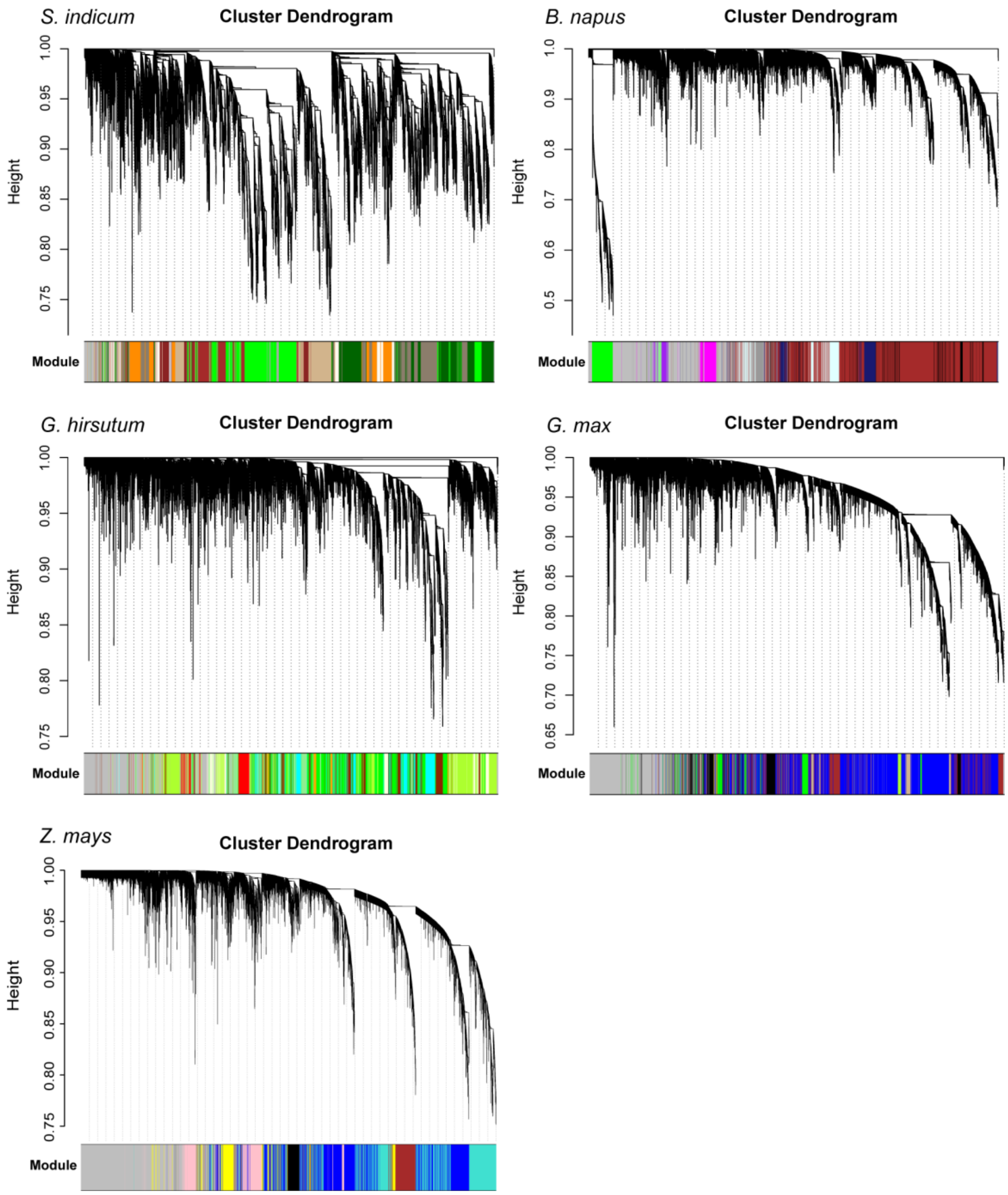

Figure S15 Hierarchical clustering trees showing coexpression modules identified using WGCNA. Modules correspond to branches and are labeled by colors as indicated by the color band "Module" underneath the tree.

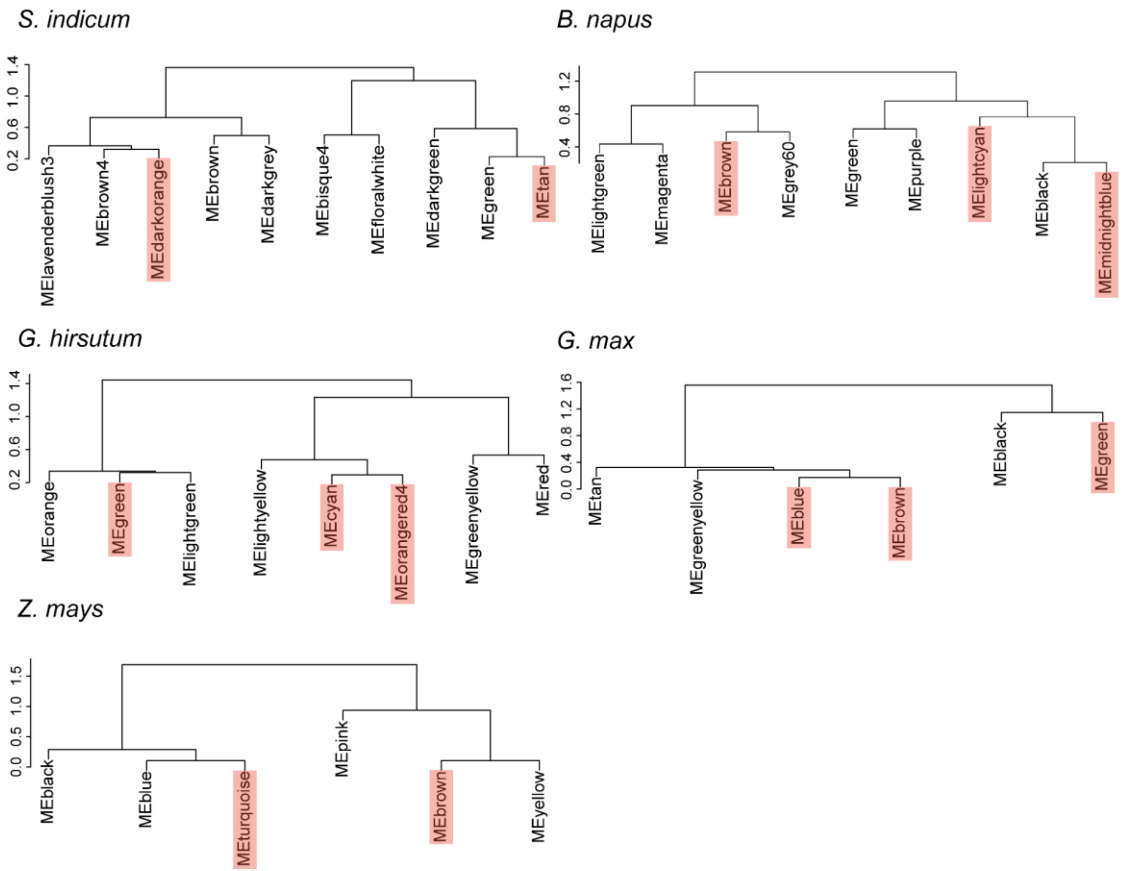

Figure S16 Hierarchical clustering dendrograms of the eigengenes of each module in which the dissimilarity of eigengenes EI, EJ is given by 1-cor(EI; EJ). The shorter the height of the clustering tree, the higher the correlation (absolute value) of the two modules. The modules with red background are the modules related to SOS identified.

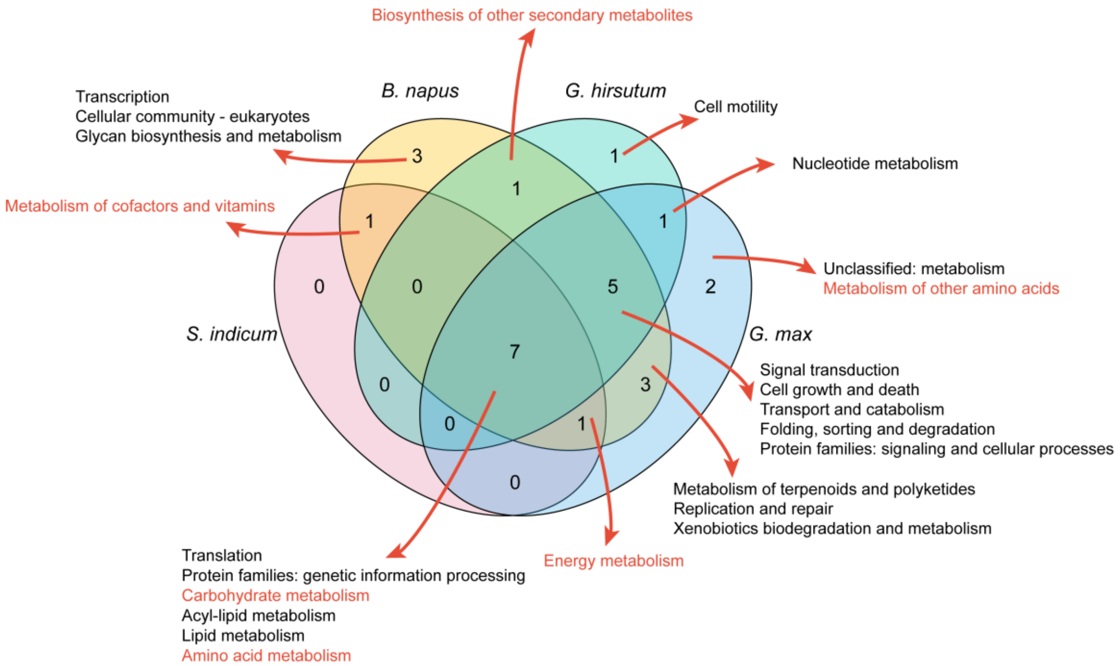

Figure S17 Venn diagram of KEGG ko2 pathways associated with the hubs of SOS modules in each oil crop (GSEA $P < 0.05$, FDR $Q < 0.1$).

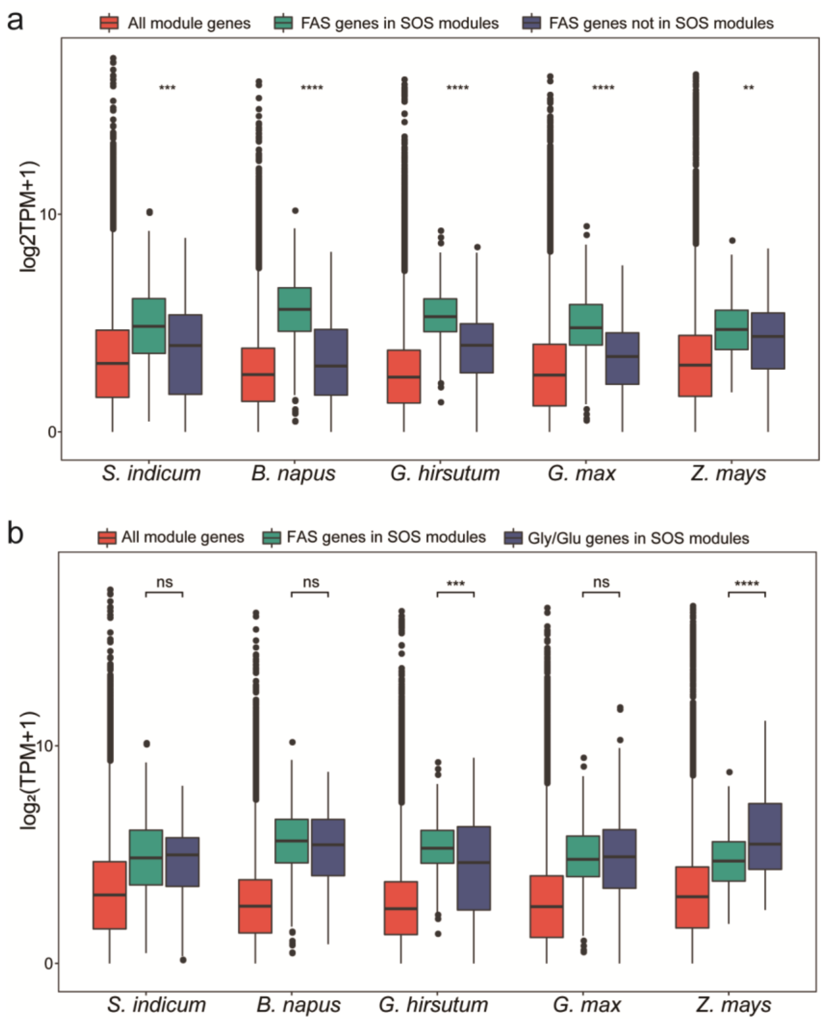

Figure S18 Comparison of expression levels between pathway genes in the low-oil materials. (a) Comparison of expression levels between FAS genes in SOS modules and FAS genes not in the SOS modules in low-oil materials of each crop. (b) Comparison of expression levels between FAS genes in SOS modules and 'Glycolysis / Gluconeogenesis' genes in the SOS modules in low-oil materials of each crop. ns *P* value > 0.05; * *P* value ⩽ 0.05; ** *P* value ⩽ 0.01; *** *P* value ⩽ 0.001; **** *P* value ⩽ 0.0001, Wilcoxon rank-sum test.

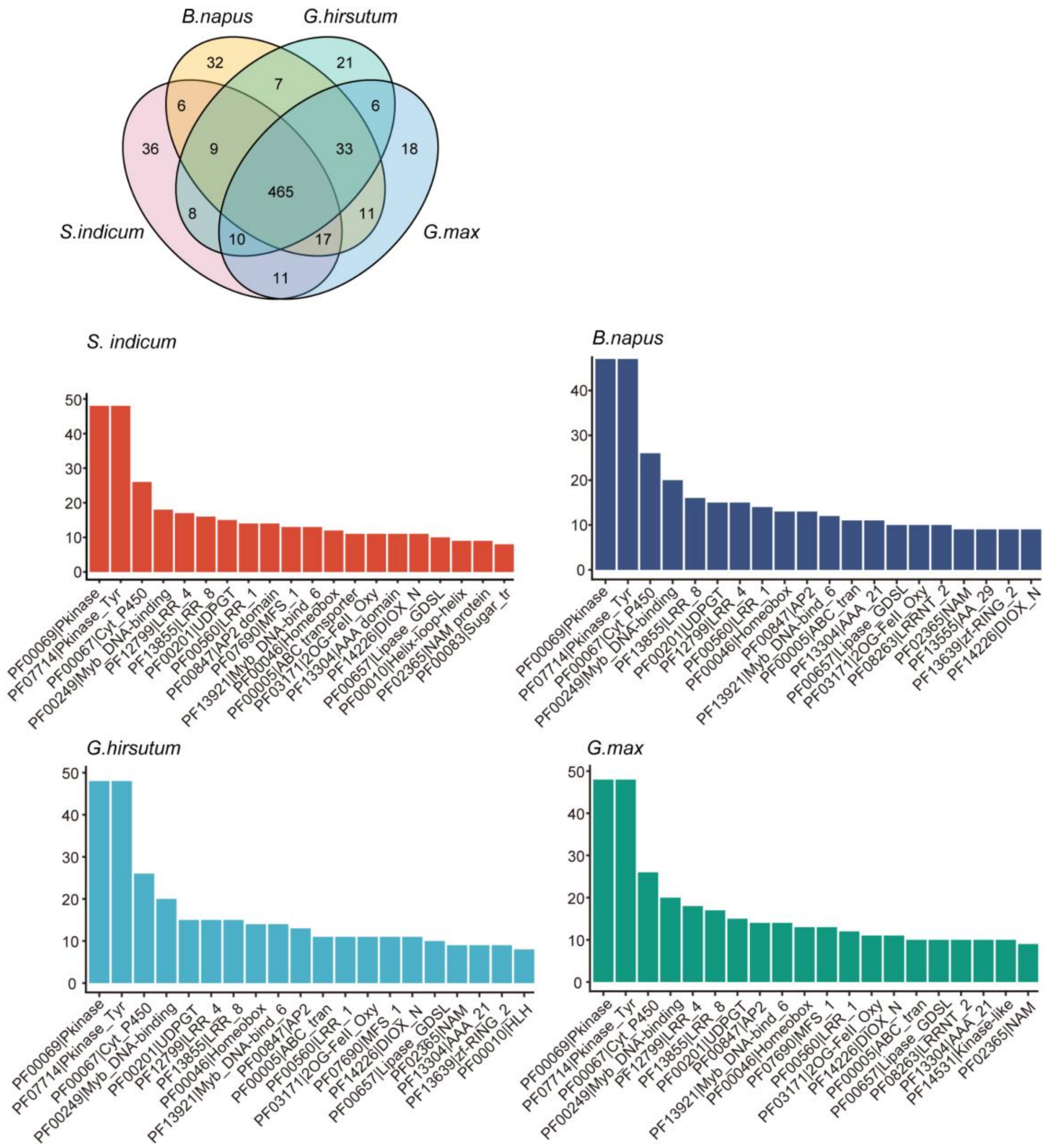

Figure S19 Venn diagram of the protein domain PFAM annotations of 692 aligned genes in conserved networks and the top 20 PFAM annotations in counts in each species.

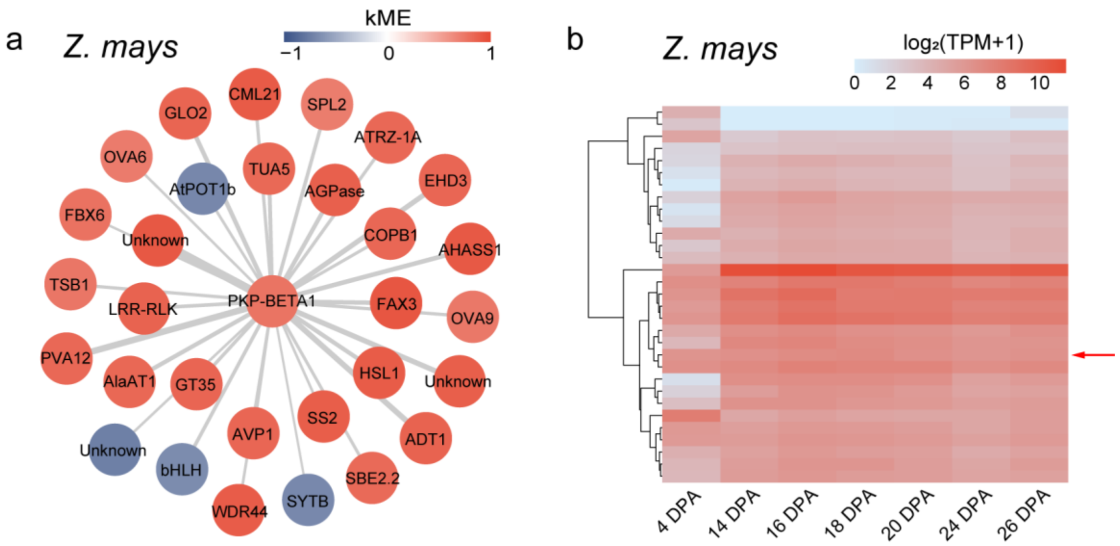

Figure S20 The *PK* network in maize and its expression profile. (a) The top 30 genes in weights connected to the *PK*. The node color represents the kME value of the corresponding gene in the network. (b) Heatmap of the *PK* and the top 30 genes in weights connected to the *PK*. Red arrows point to the *PK* in maize.
